# Beyond binding change: the molecular mechanism of ATP hydrolysis by F_1_-ATPase and its biochemical consequences

**DOI:** 10.3389/fchem.2023.1058500

**Published:** 2023-05-30

**Authors:** Sunil Nath

**Affiliations:** Department of Biochemical Engineering and Biotechnology, Indian Institute of Technology Delhi, Hauz Khas, New Delhi, India

**Keywords:** bioenergetics, ATP theory and mechanism, consistency with physical laws, Boyer's binding change mechanism of ATP synthesis/hydrolysis, Nath's torsional mechanism of energy transduction and ATP synthesis/hydrolysis and two-ion theory of energy coupling, molecular motors, mode of action of F1-ATPase inhibitors, ligand displacement/substitution, ADP-ATP exchange

## Abstract

F_1_-ATPase is a universal multisubunit enzyme and the smallest-known motor that, fueled by the process of ATP hydrolysis, rotates in 120^o^ steps. A central question is how the elementary chemical steps occurring in the three catalytic sites are coupled to the mechanical rotation. Here, we performed cold chase promotion experiments and measured the rates and extents of hydrolysis of preloaded bound ATP and promoter ATP bound in the catalytic sites. We found that rotation was caused by the electrostatic free energy change associated with the ATP cleavage reaction followed by Pi release. The combination of these two processes occurs sequentially in two different catalytic sites on the enzyme, thereby driving the two rotational sub-steps of the 120^o^ rotation. The mechanistic implications of this finding are discussed based on the overall energy balance of the system. General principles of free energy transduction are formulated, and their important physical and biochemical consequences are analyzed. In particular, how exactly ATP performs useful external work in biomolecular systems is discussed. A molecular mechanism of steady-state, trisite ATP hydrolysis by F_1_-ATPase, consistent with physical laws and principles and the consolidated body of available biochemical information, is developed. Taken together with previous results, this mechanism essentially completes the coupling scheme. Discrete snapshots seen in high-resolution X-ray structures are assigned to specific intermediate stages in the 120^o^ hydrolysis cycle, and reasons for the necessity of these conformations are readily understood. The major roles played by the “minor” subunits of ATP synthase in enabling physiological energy coupling and catalysis, first predicted by Nath's torsional mechanism of energy transduction and ATP synthesis 25 years ago, are now revealed with great clarity. The working of nine-stepped (bMF_1_, hMF_1_), six-stepped (TF_1_, EF_1_), and three-stepped (PdF_1_) F_1_ motors and of the α_3_β_3_γ subcomplex of F_1_ is explained by the same unified mechanism without invoking additional assumptions or postulating different mechanochemical coupling schemes. Some novel predictions of the unified theory on the mode of action of F_1_ inhibitors, such as sodium azide, of great pharmaceutical importance, and on more exotic artificial or hybrid/chimera F_1_ motors have been made and analyzed mathematically. The detailed ATP hydrolysis cycle for the enzyme as a whole is shown to provide a biochemical basis for a theory of “unisite” and steady-state multisite catalysis by F_1_-ATPase that had remained elusive for a very long time. The theory is supported by a probability-based calculation of enzyme species distributions and analysis of catalytic site occupancies by Mg-nucleotides and the activity of F_1_-ATPase. A new concept of energy coupling in ATP synthesis/hydrolysis based on fundamental ligand substitution chemistry has been advanced, which offers a deeper understanding, elucidates enzyme activation and catalysis in a better way, and provides a unified molecular explanation of elementary chemical events occurring at enzyme catalytic sites. As such, these developments take us beyond binding change mechanisms of ATP synthesis/hydrolysis proposed for oxidative phosphorylation and photophosphorylation in bioenergetics.

## 1 Introduction

The F_O_F_1_–ATP synthase catalyzes the ATP synthesis/hydrolysis reaction, vital to life in all living organisms (Penefsky, 1985; Boyer, 1993; Abrahams et al., 1994; Weber and Senior, 1997; Allison, 1998; Nath, 2002; Nath, 2003; Adachi et al., 2007; Nath, 2008; Nath and Nath, 2009; Nakano et al., 2022). It contains a hydrophilic F_1_ moiety that lies ∼4.5 nm above the surface of the membrane containing a hydrophobic F_O_ sector connected by two ∼1-nm-thick central and peripheral stalks (Abrahams et al., 1994; Aggeler et al., 1997; Nath, 2002; Nakano et al., 2022). The headpiece of F_1_ houses three β-catalytic sites, whereas the membrane-bound F_O_ contains access pathways that couple ion translocation to conformational changes of catalytic sites in F_1_ (Abrahams et al., 1994; Aggeler et al., 1997; Noji et al., 1997; Nath, 2002; Nath, 2003; Adachi et al., 2007; Nath, 2008; Martin et al., 2015).

The *Escherichia coli* enzyme, containing eight different subunits, is considered a prototype for ATP synthases from different organisms. The isolated F_1_ sector is an ATPase consisting of five subunits (α, β, γ, δ, and ε) with a conserved subunit stoichiometry α_3_β_3_γ δε in all organisms. In *E. coli* F_1_, the molecular masses of α, β, γ, δ, and ε measure 55.3, 50.3, 31.6, 19.3, and 14.9 kDa, respectively (Weber and Senior, 1997).

The reaction mechanism of the hydrolysis of ATP in a catalytic site of the soluble F_1_ (Weber and Senior, 1997; Allison, 1998; Nath, 2003; Adachi et al., 2007; Nath, 2008; Nath and Nath, 2009) or membrane-bound F_O_F_1_ (Penefsky, 1985; Boyer, 1993; Nath, 2008; Nath and Nath, 2009; Nakano et al., 2022) of the ATP synthase can be described by the following elementary kinetic steps:

$$F_{1}+MgATP\to F_{1}.MgATP\to F_{1}.MgADP.Pi\to F_{1}.MgADP+Pi\to F_{1}+MgADP.$$ (1)

The first step represents the formation of the enzyme–substrate complex, the second step is the catalytic step, and steps 3 and 4 describe the sequential release of products. The ATP hydrolysis rate can be readily monitored by stopped/quench flow kinetic techniques.

The enzyme complex contains three catalytic sites located primarily on the β-subunits of the F_1_ portion at the α–β interface (Abrahams et al., 1994; Weber and Senior, 1997; Nath, 2002; Nakano et al., 2022), which work together during multisite hydrolysis (Adachi et al., 2007; Nath, 2008). It has now been conclusively established that a domain consisting of the “minor” γ- and ε-subunits rotates relative to the α_3_β_3_ hexamer during ATP hydrolysis and synthesis. The rotation, inferred first from biochemical crosslinking studies (Aggeler et al., 1997), has been visualized directly using epifluorescence microscopy during ATP hydrolysis by the F_1_-ATPase (Noji et al., 1997) and during ATP synthesis (Martin et al., 2015).

Although the direct link between catalytic site events and rotation has been confirmed, no unequivocal correlation has been established between the rate of rotation of the γ–ε domain and the kinetics of the individual steps in the ATP hydrolysis (Eq. 1) and ATP synthesis reactions. The analysis is greatly complicated by the fact that ATP can bind in three β-catalytic sites that are characterized by high (site 1), intermediate (site 2), and low (site 3) affinity for nucleotides. Thus, *a priori*, the driving force for rotation during steady-state V_max_ ATP hydrolysis could be the binding energy of ATP and/or the free energy change associated with the ATP cleavage reaction and product release in any of these three catalytic sites. Boyer’s binding change mechanism (Boyer et al., 1973; Boyer, 1993) and Nath's torsional mechanism of ATP synthesis/hydrolysis (Nath, 2002; Nath, 2008; Mehta et al., 2020) are two important and detailed theories that have been proposed to explain the functioning of the enzyme during steady-state ATP synthesis/hydrolysis. Other physical models of F_1_-ATPase have been developed by various theory groups (Wang and Oster, 1998; Bai et al., 2020; Gerritsma and Gaspard, 2010; Lenz et al., 2003; Mukherjee and Warshel, 2011; Volkán-Kacsó and Marcus, 2017; Nam and Karplus, 2019; Volkán-Kacsó and Marcus, 2022). These latter works, though important in their own right, do not address the biochemical issues of “unisite” catalysis, cold chase, and rate enhancement in multisite catalysis when the substrate binds to additional catalytic sites. However, we agree with the reviewer that greater attention should be given to theory and that the right theory has the power to catalyze rapid progress in bioenergetics and several interdisciplinary fields of biology.

Functioning in these β-catalytic sites can be biochemically differentiated because ATP hydrolysis in the high-affinity catalytic site 1 can be monitored by so-called “unisite” catalysis measurements with sub-stoichiometric amounts of [γ-^32^P]ATP relative to F_1_ (Penefsky, 1985). These conditions lead to preferential binding of the substrate in a single site ( i.e., in the high-affinity site 1), resulting in the formation of the enzyme–substrate complex in the site (Step 1 in Eq. 1). Therefore, it can be distinguished from nucleotide binding into lower affinity catalytic sites (preferentially site 2, and with a far lower probability of filling the least affinity site 3), which causes product release from the high-affinity site 1 in cold chase experiments (Penefsky, 1985; García and Capaldi, 1998). Such equilibrium and kinetic experiments can help elucidate the mechanism of ATP hydrolysis by F_1_-ATPase.

The chemical reactions of “unisite” catalysis shown in Eq. 1 characterize a single turnover event. However, whether all the elementary steps of the reaction scheme take place in a single catalytic site [site 1, or T, as hypothesized by Penefsky (1985)], in site 2 (i.e., in L), or in both has not been conclusively established. Furthermore, does the catalytic conformation of site 1 (T) need to be altered to site 2 (L), as proposed previously (Nath, 2003; Nath, 2008), to enable ATP hydrolysis occurrence in the “unisite” mode described previously (Penefsky, 1985) or in its transition to multisite catalysis (Nath, 2008)? There has been no report on “unisite” ATP synthesis to date. Therefore, what exactly is “unisite” ATP hydrolysis? Under what special conditions does it occur? What is the biochemical basis underlying “unisite” catalysis? Can its relationship to steady state (multisite) ATP hydrolysis be characterized and can the rate enhancement in the progress from “unisite” to multisite hydrolysis be understood mechanistically? These problems have been considered “elusive” in recent work (Nakano et al., 2022).

A major reason for the difficulty and elusiveness of the problems stated above in ATP hydrolysis by F_1_-ATPase arises from their concatenated nature. A detailed solution of the molecular mechanism of steady-state ATP hydrolysis by F_1_-ATPase is required to fully understand turnover events underlying “unisite” catalysis (Penefsky, 1985; Nakano et al., 2022), cold chase (García and Capaldi, 1998), and progression to multisite hydrolysis (Weber and Senior, 1997; Adachi et al., 2007; Nath, 2008). Such a unified mechanism of ATP synthesis/hydrolysis has already been formulated (Nath, 2008). Can it help solve the problem? Looking at the problem from another angle, experiments in unisite and cold chase ATP hydrolysis can offer novel insights into steady-state multisite ATP hydrolysis by F_1_-ATPase. How do models and mechanisms of ATP hydrolysis perform with respect to these experiments? Can the models be refined in light of these experimental results?

A definitive solution to the aforementioned longstanding “elusive” problems is attempted in this work. The refined molecular mechanism of ATP hydrolysis by F_1_-ATPase helps interpret X-ray structural snapshots, especially those close to the ATP-waiting state at 0^o^ (or 120^o^) in the catalytic cycle, and assign them to specific conformations of the enzyme during catalysis.

This article is organized as follows. Section 2 describes the experimental methods used. Section 3.1 reports data on the rates and extents of hydrolysis of preloaded bound ATP and promoter ATP in cold chase promotion experiments. Mechanistic implications arising from steady-state ATP hydrolysis by F_1_-ATPase are deduced in Section 3.2. This enables the formulation of general principles for biological free energy transduction with its manifold physical and biochemical consequences, which are analyzed in Section 3.3. A molecular mechanism of steady-state, trisite ATP hydrolysis by F_1_-ATPase consistent with physical laws and principles and the body of available biochemical information that goes beyond previous theories (Boyer et al., 1973; Boyer, 1993) is formulated in Section 3.4. The structural and biochemical consequences of the new molecular vistas are presented in Section 4. In particular, the central role of the γ-subunit and especially of the ε-subunit as conduits in energy coupling, that enable fine-tuned conformational changes of the β-catalytic sites essential to catalysis in ATP synthesis/hydrolysis by F_O_F_1_-ATP synthase/F_1_-ATPase, are discussed. The working of nine-stepped (bMF_1_, hMF_1_), six-stepped (TF_1_, EF_1_), and three-stepped (PdF_1_) F_1_ motors and of the α_3_β_3_γ subcomplex of F_1_ is explained by a unified mechanism. The theory is supported by a probability-based calculation of enzyme species distributions and analysis of catalytic site occupancies by Mg-nucleotides and the activity of F_1_-ATPase. Some novel predictions of the unified theory that are of pharmacological importance are also made in Section 4. A new concept of energy coupling in ATP synthesis/hydrolysis based on fundamental *ligand substitution chemistry* is proposed, which takes us beyond the binding change mechanism of ATP synthesis/hydrolysis.

A mathematical model for estimating economics and opportunity cost in choosing between competing theories is developed in the Supplementary Section.

## 2 Methods

MF_1_ was prepared from bovine heart mitochondria using standard procedures, as described previously (Penefsky, 1979). The specific activity of the enzyme was 95 units/mg. The Mg buffer contained 40 mM Tris-MES, 0.25 M sucrose, and 0.5 mM MgSO_4_, with a pH of 8.0 at 23°C. The Pi concentration was kept constant at 2 mM in the reaction mixture. Aliquots of an ammonium sulfate suspension of MF_1_ were centrifuged, and the pellets were separated from the (NH_4_)_2_SO_4_ supernatant. The pellets were dissolved in 100 μL Mg buffer, and the enzyme solution was passed through a centrifuge column equilibrated with the same buffer. The enzyme was incubated with Mg buffer for 1 h at 23°C. [γ-^32^P]ATP was prepared as described previously (Glynn and Chappell, 1964). The specific activity of the [γ-^32^P]ATP used was 10^6^–10^7^ counts min^–1^ nmol^–1^. Equilibrium chase promotion experiments were performed in 1 ml reaction mixtures with magnetic stirring, and kinetic rate promotion experiments were performed in a quenched flow apparatus (Penefsky, 1985). The error bars arise in part from a small percentage in [γ-^32^P]ATP solutions that are unreactive with hexokinase (∼2%–3%).

A measure of 100 μL of a solution containing 1 nmol MF_1_ in Mg buffer was added to a glass reaction vessel containing Mg buffer to determine the maximum equilibrium hydrolysis of preloaded and promoter ATP in cold chase experiments. The solution was stirred at high speed with a magnetic stirrer and stirrer bar. A volume of 20 μL of 15 μM [γ-^32^P]ATP or ATP was pipetted and incubated for 3 s. Chase solutions were pipetted from 0.5 mM stock solutions of ATP or [γ-^32^P]ATP, respectively. The final volume measured 1.0 ml in Mg buffer. After the addition of the chase, the solutions were incubated for 10 s, and the reaction was thereafter terminated by adding 0.2 ml of 70% perchloric acid. For the control without adding promoter ATP, the reaction was allowed to continue for 10 s and quenched by adding perchloric acid before the chase. ^32^Pi was separated from [γ-^32^P]ATP and counted as described (Penefsky, 1985), and results are expressed as percent hydrolysis of added [γ-^32^P]ATP.

For determining the kinetic rate of hydrolysis of preloaded, bound [γ-^32^P]ATP, and promoter [γ-^32^P]ATP in the cold chase experiments, equal volumes of 3 μM MF_1_ in Mg buffer and 1 μM [γ-^32^P]ATP or ATP were first mixed for 2 s to allow the formation of the enzyme–substrate complex. Then, the contents were mixed with 15 μM chase ATP or [γ-^32^P]ATP, and the chased reaction mixtures passed into a vessel containing 0.8 ml Mg buffer, 0.2 ml of 70% perchloric acid, and 0.1 ml of 100 mM ATP. The residence time is the time between mixing of the MF_1_-^32^P enzyme–substrate complex with the chase ATP and quenching in acid. The reference point of zero time was obtained by directly collecting the solution containing the enzyme–substrate complex into the perchloric acid quench without the chase. ^32^Pi formed was isolated and counted as described (Penefsky, 1985), and the data were plotted as percent hydrolysis of added [γ-^32^P]ATP.

F_1_ and [γ-^32^P]ATP were mixed at 1) 0.5 μM each and 2) 1 μM each in Mg buffer at 23°C. In other experiments, 10% excess F_1_ was employed with respect to the substrate to obtain the distribution of bound substrate and product at F_1_-ATPase catalytic sites. A complex between the two species was allowed to form, and unbound ^32^P was removed on centrifuge columns. Column effluents were collected at 1, 5, 10, and 15 min in perchloric acid quench, the bound ^32^Pi was determined, and the fraction f of total bound ^32^P present as ^32^Pi was quantitated at various times.

## 3 Results

### 3.1 Rates and extents of hydrolysis of bound [γ-^32^P]ATP and chase [γ-^32^P]ATP in promotion experiments

As shown in step 1 of the reaction scheme (Eq. 1), incubation of 1 μM MF_1_ with 0.3 μM [γ-^32^P]ATP, i.e., under sub-stoichiometric conditions that predominantly favor substrate binding to a single catalytic site of the F_1_-ATPase [site 1, the tight (T) site with the highest affinity for ATP)], results in the formation of an enzyme–substrate complex. This is followed by the hydrolysis of ATP to ADP.Pi on the enzyme, limited by the slow rate of dissociation of the products (Eq. 1). The first line of Table 1 shows that the percentage of hydrolysis of added [γ-^32^P]ATP after 10 s incubation time is only 30%. Addition as a cold chase of 5–20 μM of promoter ATP (that binds predominantly to a second catalytic site of the enzyme, i.e., site 2, the loose (L) site with intermediate affinity for ATP) results in hydrolysis of 92%–96% of [γ-^32^P]ATP bound in the highest affinity catalytic site 1. Similar results were obtained when 3 mM ATP is used as the chase when all the three catalytic sites of F_1_ are expected to be occupied by bound nucleotide. Note that a super-stoichiometric concentration of promoter ATP is essential to ensure that the F_1_-ATPase undergoes multiple cycles of rotation during the 10 s incubation time, given that a major objective of the work is to detect the rate enhancement (over unisite rates) that increases turnover to V_max_ when ATP is allowed to bind at multiple catalytic sites. However, complete (i.e., 100%) hydrolysis of the added ATP was not observed even with mM concentrations of the cold chase. Table 1 shows that when normal ATP is bound in the highest affinity site 1 (T) and 5–20 μM radioactive [γ-^32^P]ATP is used as the chase, 92%–94% of the promoter ATP is hydrolyzed in site 2 (L) in the same 10 s time period.

**TABLE 1:** Maximum extent of hydrolysis of preloaded, bound [γ-^32^P]ATP and promoter [γ-^32^P]ATP during a cold chase experiment (mean ± SD).

Concentration of promoter ATP (μM)	Hydrolysis of loaded [γ-^32^P]ATP (%)	Hydrolysis of promoter [γ-^32^P]ATP (%)
0	30 ± 2	0
5	92 ± 2	92 ± 3
10	92 ± 2	93 ± 3
15	95 ± 2	94 ± 3
20	96 ± 2	94 ± 3

**FIGURE 1:**
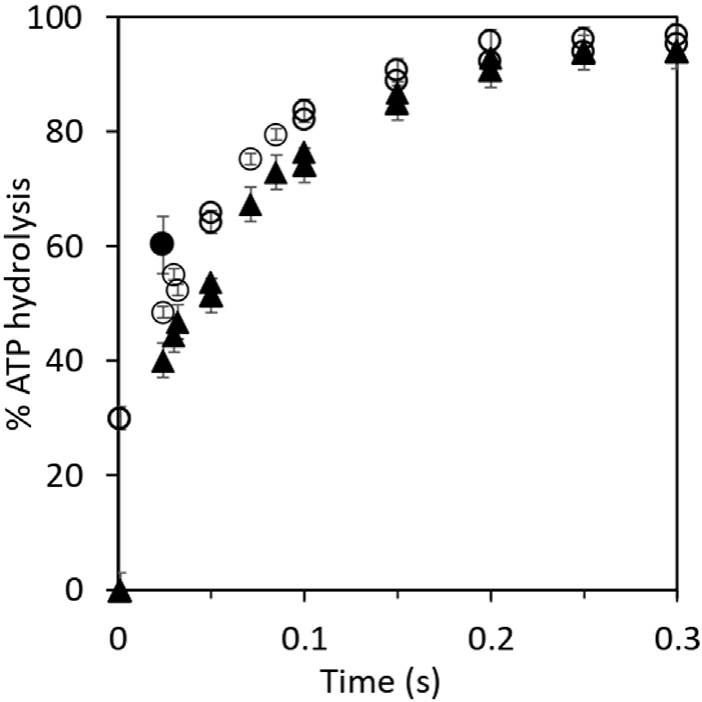
Kinetics of hydrolysis of bound [γ-^32^P]ATP and of promoter [γ-^32^P]ATP in cold chase experiments. Equal volumes of 3 μM MF_1_ in Mg buffer and 1 μM [γ-^32^P]ATP or ATP were first mixed for 2 s before the addition of the chase. (O) Rates of hydrolysis of 3 μM MF_1_ in Mg buffer and 1 μM preloaded, bound [γ-^32^P]ATP with 15 μM chase ATP. The final concentrations in the chased reaction mixtures measured 1 μM MF_1_, 0.3 μM [γ-^32^P]ATP, and 5 μM cold chase ATP. (▲) Rates of hydrolysis of 15 μM chase (promoter) [γ-^32^P]ATP with 3 μM MF_1_ in Mg buffer and 1 μM preloaded ATP. The final concentrations in the chased reaction mixtures measured 1 μM MF_1_, 0.3 μM ATP, and 5 μM cold chase [γ-^32^P]ATP. The bold circle (●) shows the observation when the promoter ATP concentration was increased from 5 μM to 3 mM. The unisite MF_1_.^32^P complex was mixed with a large excess of nonradioactive MgATP (final concentration of 3 mM in the chase), and the reaction was allowed to proceed for 20 ms before injection into perchloric acid quench. By the next temporal assay point of 50 ms, almost complete hydrolysis (>95%) of [γ-^32^P]ATP had already occurred at 3 mM promoter ATP concentration. This data point is not plotted on the graph because complete hydrolysis could, in principle, have occurred at any time between 20 and 50 ms at 3 mM promoter [ATP].

The aforementioned results were examined further by kinetic analysis, and the results are plotted in Figure 1. The 30% residual hydrolysis of preloaded [γ-^32^P]ATP at the end of the 2 s incubation period is plotted as the zero time value on the *y*-axis for the upper curve before the addition of chase ATP. The final concentrations in the chased reaction mixtures were 1 μM F_1_, 0.3 μM [γ-^32^P]ATP, and 5 μM cold chase ATP (Figure 1). In the case of the lower curve, normal ATP was loaded in the highest affinity catalytic site, and [γ-^32^P]ATP was used as the chase (Figure 1). This enabled measurement of the rate and extent of the hydrolysis of chase ATP under the same conditions as for the upper curve. The features of the progress curve of hydrolysis of the chase ATP bound in site 2 (lower curve) are kinetically similar to those of the hydrolysis of ATP bound in the highest affinity catalytic site 1 (upper curve), as long as well-mixed conditions were ensured in the experiments. This observation requires separate interpretation and discussion (Sections 3.2, 3.4). Under the chosen experimental conditions and in the presence of rapid and efficient mixing of the solutions, both progress curves in Figure 1 are well characterized by a first-order rate constant measuring 12–15 s^–1^, given the experimental errors. Data on the rapid hydrolysis of [γ-^32^P]ATP in site 1 when promoter ATP concentration was increased from 5 μM to 3 mM are also shown in Figure 1.

### 3.2 Mechanistic implications

The results described in Section 3.1 have several mechanistic implications for ATP hydrolysis by F_1_-ATPase. Bullough et al. (1987) had previously performed experiments in which ATP bound in the highest affinity catalytic site of F_1_-ATPase appeared to hydrolyze severalfold slower than ATP added as a promoter. Based on these observations, the authors suggested that the highest affinity site 1 is not a normal catalytic site on F_1_ (Bullough et al., 1987). However, we did not observe such a rate discrepancy between the two promotion experiments. The experimental results in Table 1 and Figure 1 show that the enzyme molecules undergoing single turnover events of slow “unisite” catalysis (on the order of 0.1 s^–1^) are recruited into the “normal” catalytic pathway of F_1_-ATPase during rapid multisite V_max_ hydrolysis of at least 100 s^–1^ by the addition of excess ATP, and as a result, the unisite characteristics of F_1_ are amalgamated. Furthermore, the bound ATP in site 1 is hydrolyzed during the chase at approximately the same rate as the chase ATP bound at a catalytic site with intermediate affinity and not at substantially lower rates as reported (Bullough et al., 1987). We estimate that ATP bound in the catalytic sites is hydrolyzed at V_max_ rates of >100 s^–1^ at promoter concentrations of 5 μM since the shortest residence time in our experiments is 20 ms. Under these conditions, 20% hydrolysis represents at least one enzyme turnover. Similar results were obtained when 3 mM ATP was used in the cold chase, except that the V_max_ rates were higher (Figure 1). In this experiment, the unisite MF_1_.^32^P complex formed previously was mixed with a large excess of normal MgATP at a final concentration of 3 mM. The reaction was allowed to proceed for 20 ms before quenching into perchloric acid. The promoted hydrolysis occurred very rapidly within this chase time period, as shown by the bold circle, and hydrolysis of [γ-^32^P]ATP was essentially complete by the next temporal measurement point of 50 ms. The results shown in Table 1 and Figure 1 raise a question: how do two different catalytic sites on the multisubunit F_1_ enzyme that are spatially distant away from each other (and purportedly possess different affinities for binding ATP) hydrolyze ATP at approximately the same rates and with similar kinetics (the following paragraph and Section 3.4)?

The cold chase experimental results have an even more important biological implication: they show that two catalytic sites on F_1_ (at different times) can hydrolyze ATP at the so-called highest affinity site 1 (T) (Penefsky, 1985; García and Capaldi, 1998) and at the intermediate affinity site 2 (L). This important conclusion arises from the analysis given the two progress curves/rows in Figure 1 and Table 1 of the hydrolysis of [γ-^32^P]ATP when preloaded in site 1 or when bound as a promoter in site 2. If the conformational change (∼80° rotation of the central γ-subunit of the enzyme) due to binding and subsequent hydrolysis of ATP at site 2 causes site 1 (T) to be converted to (a new) site 2 (L), then similar rates and extents of ATP hydrolysis in the two cases are also logically explained. In other words, the two sites are the same: both are L-sites (site 2). In other words, only site 2 can hydrolyze ATP and release products.

**FIGURE 2:**
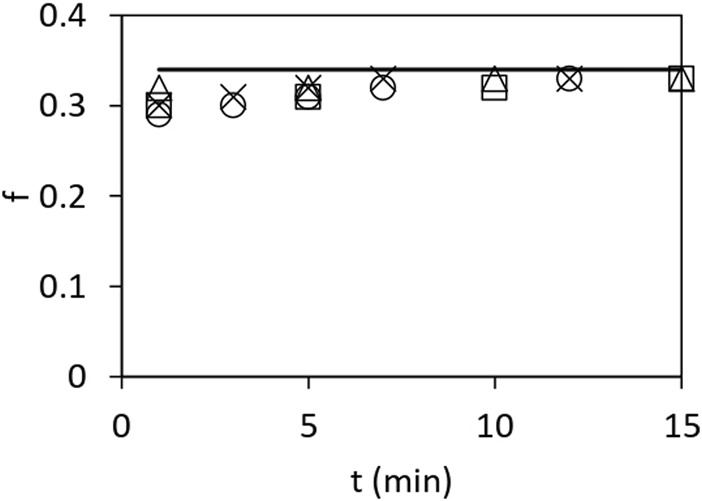
Fraction f of bound ^32^Pi to total bound ^32^P as a function of time after mixing mitochondrial F_1_ and [γ-^32^P]ATP. Enzyme and substrate concentrations of 0.5 μM each (□), 1 μM each (∆), with 10% excess enzyme concentration over 0.5 μM substrate (○), and with 10% excess enzyme concentration over 1 μM substrate (×). The data are bounded from above by the bold horizontal line given by f=0.333 .

Figure 2 shows the ratio f of bound ^32^Pi to total bound ^32^P at two different concentrations of F_1_ and [γ-^32^P]ATP and various incubation times from 1 to 15 min. This ratio was approximately constant at one-third under all conditions tested; i.e., the data shown in Figure 2 are bounded from above by the line f=0.333 . Similar results were obtained when 10% excess F_1_ over substrate was used. It should be clearly understood that the total bound ^32^P includes both bound ^32^Pi and bound [γ-^32^P]ATP species. The results can be either interpreted in terms of an equilibrium constant for reversible hydrolysis or explained equally well by a quasi-steady state constant for irreversible hydrolysis in which f=0.333 remains constant for any mode of catalysis (e.g., trisite catalysis); i.e., f defines a characteristic kinetic property of the system.

In summary, it is not sufficient that the F_1_-ATPase simply binds ATP in site 2 (L); the enzyme needs to additionally hydrolyze the bound ATP (that had exchanged with ADP in site 2 (L)) (Nath, 2008) to ADP.Pi in site 2, after which Pi needs to leave site 2 (L) to induce rotation by the chase ATP. Thus, after bond cleavage due to ATP hydrolysis on the enzyme and the reduction in binding of Pi, it is the progressive moving away of the Pi from bound MgADP (Nath and Nath, 2009) in site 2 (L) and the firing of Pi into the solution to infinity that donates energy and is responsible for the rotation of γ, whereupon the L site changes to a closed (C) site. This primary clockwise rotation of the top of γ (viewed from the F_1_ side) also changes the conformation of site 1 (T) to a new site 2 (L), after which the bound ATP hydrolyzes in the new site 2 (L) to ADP.Pi, and Pi subsequently leaves and donates energy for a further ∼40° rotation, as described in great detail previously (Nath, 2008), and mathematically modeled using basic electrostatic principles (Nath and Nath, 2009) (Section 3.4). The new aspect is contained in the key insight that ATP hydrolysis and Pi release [not ATP binding as in previous theories of free energy transduction (Boyer et al., 1973; Boyer, 1993)] in site 2 are required to explain the chase promotion experiments because otherwise, no ^32^Pi counts should have been registered. This has major biological implications and permits us to formulate general physical principles for free energy transduction.

### 3.3 General physical principles of energy transduction and biochemical consequences: how does ATP perform useful external work?

According to the basic tenets of the binding change mechanism (Boyer et al., 1973; Boyer, 1993), the principal energy-releasing step in F_1_-ATPase, muscle contraction, and other processes utilizing ATP is the one accompanying ATP binding, with hydrolysis merely serving for release of ADP and Pi. The mechanism proposed that ATP binds very tightly in site 1 (T), with a very low dissociation constant K_d_—vis-à-vis site 2 (L) such that ATP is differentially stabilized on the enzyme surface relative to ADP + Pi by ≥ 60 kJ/mol. Hence, the mechanism proposed that a catalytic site shows reversible ATP synthesis/hydrolysis with an equilibrium constant K_eq_ close to 1. Historically, these concepts have greatly influenced the interpretation of experimental measurements and catalytic mechanism in F_1_-ATPase and muscle myosin. However, are these concepts correct?

As discussed previously, a highly sequestered catalytic site was required as ATP synthesis is believed to occur with free reversal of ATP hydrolysis on the enzyme (Boyer, 1993). Earlier measurements of a K_d_ of 1 pM for catalytic site 1 (Penefsky, 1985) seemed apparently consistent with the aforementioned suggestion because, with a typical K_d_ value of 0.5 μM for site 2, this corresponded to a difference in the binding energy of $RTln\left(\frac{0.5\times 10^{-6}}{1\times 10^{-12}}\right)=33.8$ kJ/mol, slightly less than the standard free energy change of ATP hydrolysis of ∼36 kJ/mol under physiological conditions (Phillips et al., 1969; Rosing and Slater, 1972; Nath and Nath, 2009), though still greatly short of the complete thermodynamic ∆G of ∼60 kJ/mol. However, the initial expectation of a sequestered site 1 was never met. Subsequent measurements using radioactive ATP and a hexokinase/glucose trap gave a binding affinity value of K_d_ for catalytic site 1 of only 0.2 nM (Senior et al., 1992) for the *E. coli* F_1_, leading to a relative stability of enzyme-bound intermediates of $RTln\left(\frac{0.5\times 10^{-6}}{0.2\times 10^{-9}}\right)=20.2$ kJ/mol only. This is a serious shortcoming of the theory (Boyer, 1993) because expected values of the differential stabilization are not validated by experimental measurements of the binding constants, and binding energy changes are insufficient in magnitude to perform the catalysis.

New technologically advanced experiments with various nucleotides also led to similar results (Weber and Senior, 2001). We have previously hailed these advancements in the direct measurement of catalytic site binding affinities and dissociation constants in F_1_-ATPase as an experimental breakthrough [p. 73 of Nath (2002)]. These measurements were made possible by developing a genetically engineered tryptophan probe β–Trp-331 inserted into the adenine-binding subdomain of the β-catalytic sites of *E. coli* F_1_-ATPase and optically monitoring its fluorescence during steady-state catalysis. For *E. coli* F_1_-ATPase for the physiologically important conditions of Mg^2+^ in excess over ATP, these new experiments yielded values of the dissociation constants for site 1 (T), site 2 (L), and site 3 (O) of 0.02, 1.4, and 23 μM, respectively (Weber and Senior, 2001). This leads to a differential stabilization of MgATP in site 1 with respect to site 2 of $RTln\left(\frac{1.4\times 10^{-6}}{0.02\times 10^{-6}}\right)=10.9$ kJ/mol only, which is in great shortfall of the expected stabilization. Experiments with MgITP using the optical probes gave similar values for the differential stabilization of the Mg-nucleotide between sites 1 and 2. However, ATP is readily synthesized at V_max_ rates using MgITP (Weber and Senior, 2001). We consider these the best measurements of binding affinities in F_1_ catalytic sites to date. We conclude that binding energy changes are energetically not competent to carry out ATP synthesis/hydrolysis in F_1_ as per the tenets of the binding change mechanism (Boyer et al., 1973; Boyer, 1993).

**FIGURE 3:**
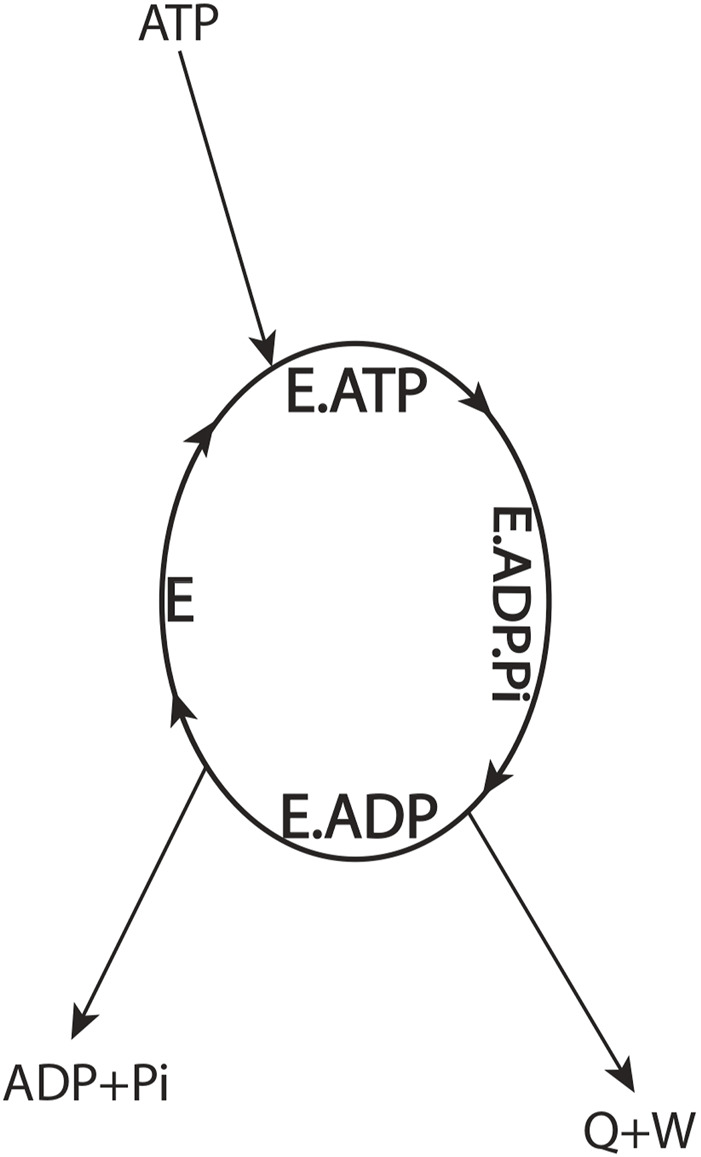
Overall energy balance for an ATP-hydrolyzing enzymatic system/biological molecular machine performing useful external work in a cyclic isothermal process (Eq. 2).

To better understand the thermodynamic aspects of the aforementioned conundrum, let us define the F_1_-ATPase or the molecular motor as the system by drawing a system boundary about the ellipse (Figure 3) and carry out an overall energy balance. For the cyclic, isothermal process mediated by the enzyme depicted by the ellipse, all thermodynamic property changes are necessarily zero. From inspection of such a diagram, we notice that binding energy changes, such as those that occur during the E+ATP→E.ATP elementary binding step, are internal to the system boundary. A general statement of the first law of thermodynamics for steady-state open systems (Figure 3) is given by the following equation, written in terms of enthalpy H as follows:

$$H_{ATP}-H_{ADP}-H_{Pi}=Q+W,$$ (2)

where the enthalpies of the species $H_{i}$ can be replaced by their internal energies $U_{i}$ if the PV work is negligible. The equation can also be written in terms of Gibbs energies $G_{i}$ instead of the enthalpies, with due consideration for the nonequilibrium nature of the process or in terms of the chemical potentials $\mu_{i}$ (Section 3.5 of Nath (2003)). As free ATP enters the system boundary in Figure 3 and free ADP and Pi leave it, Eq. 2 shows that the difference between the enthalpies or internal/electrostatic free energies of ATP and (ADP + Pi) must equal the heat released from the system Q plus work performed by the system W or equal W if heat losses are neglected.

One can argue that the first law of thermodynamics can be saved by redistribution of the overall enthalpy change from the ATP → ADP + Pi couple (Eq. 2) among the binding steps internal to the system boundary (Figure 3) to obtain Q+W . Unfortunately, the magnitude of the stabilization obtained upon redistribution of the binding state energies (only ∼10 kJ/mol) falls well short of the expected values, as shown previously. Hence, the binding energy released upon ATP binding to a catalytic site in F_1_-ATPase is insufficient for performance of the proposed magnitude of useful external work. Nath's torsional mechanism of energy transduction and ATP synthesis/hydrolysis (Nath, 2008; Nath and Nath, 2009; Mehta et al., 2020) offers a resolution of the aforementioned conundrum.

According to a basic tenet of the torsional mechanism, the energy employed for the performance of useful external work in a cyclic isothermal process must have been locked in the ATP molecule (as electrostatic potential) relative to (ADP + Pi) (Nath and Nath, 2009). The enzyme/motor serves as a key to unlocking this stored energy by the elementary step of ATP hydrolysis, and two negatively charged cleavage products (ADP and Pi) are generated. However, the electrostatic energy of these charges remains stored as potential energy, and, only after the binding of one of them (typically Pi) to the enzyme is reduced, thereby allowing the Pi to move away from bound ADP, is this potential energy made available for the performance of useful external work. Hence, only upon product Pi release can the Coulombic repulsion energy or stored potential energy of the two charges be harnessed for performing useful work. Typically, this electrostatic potential energy needs to be stored (e.g., as torsional energy or twist or as elastic strain in general) (Nath, 2008; Nath and Nath, 2009) in a region of the protein molecule by conformational changes. The protein then does useful work as it returns to its original conformation, rebinds ATP, and undergoes repeated cycles of free energy transduction.

The aforementioned general principle of energy transduction also explains why non-hydrolyzable analogs of ATP cannot perform useful external work. This primarily arises because the free energy available by reversal of the conformational change due to the binding of the ATP analog is used for the release of the bound analog. Hence, it cannot perform useful mechanical work. However, in the case of ATP hydrolysis, the products ADP and Pi are more stable in solution (relative to free ATP in solution; see Figure 3) and do not recombine. Therefore, such a reversal of the change in protein conformation is not required to release bound nucleotide. Hence, the protein conformational change can do useful work using the ATP→ADP+Pi couple.

In summary, the general physical principle emerges that the electrostatic free energy is released when ATP’s terminal P_β_–O–P_γ_ bond (γ-phosphorus–oxygen anhydride bond) is cleaved, the binding of Pi to the catalytic site is reduced, and the Pi allowed to move away to infinity from bound ADP; this energy can be used for the performance of useful work by F_1_ and by biological systems in general (Eq. 2; Figure 3).

Other differences between the binding change mechanism and the torsional mechanism have been discussed previously (Nath, 2008; Nath, 2003, and Table 1 therein). General physical principles in the membrane-bound F_O_ portion of the ATP synthase of biochemically clean reconstituted enzyme systems and during physiological steady-state ATP synthesis, such as the inviolability of electroneutrality and differences with the chemiosmotic theory (Mitchell, 1966; Mitchell, 1969; Mitchell, 1981), have been covered in earlier publications (Nath, 2010b; Nath, 2017; Nath, 2018a; Nath, 2021a; Nath, 2022a; Jain et al., 2004; Agarwal, 2011; Channakeshava, 2011; Villadsen et al., 2011; Wray, 2015; Levy and Calvert, 2021; Juretić, 2022).

### 3.4 Detailed molecular mechanism of ATP hydrolysis by F_1_-ATPase consistent with the biochemical observations and the physical laws

**FIGURE 4:**
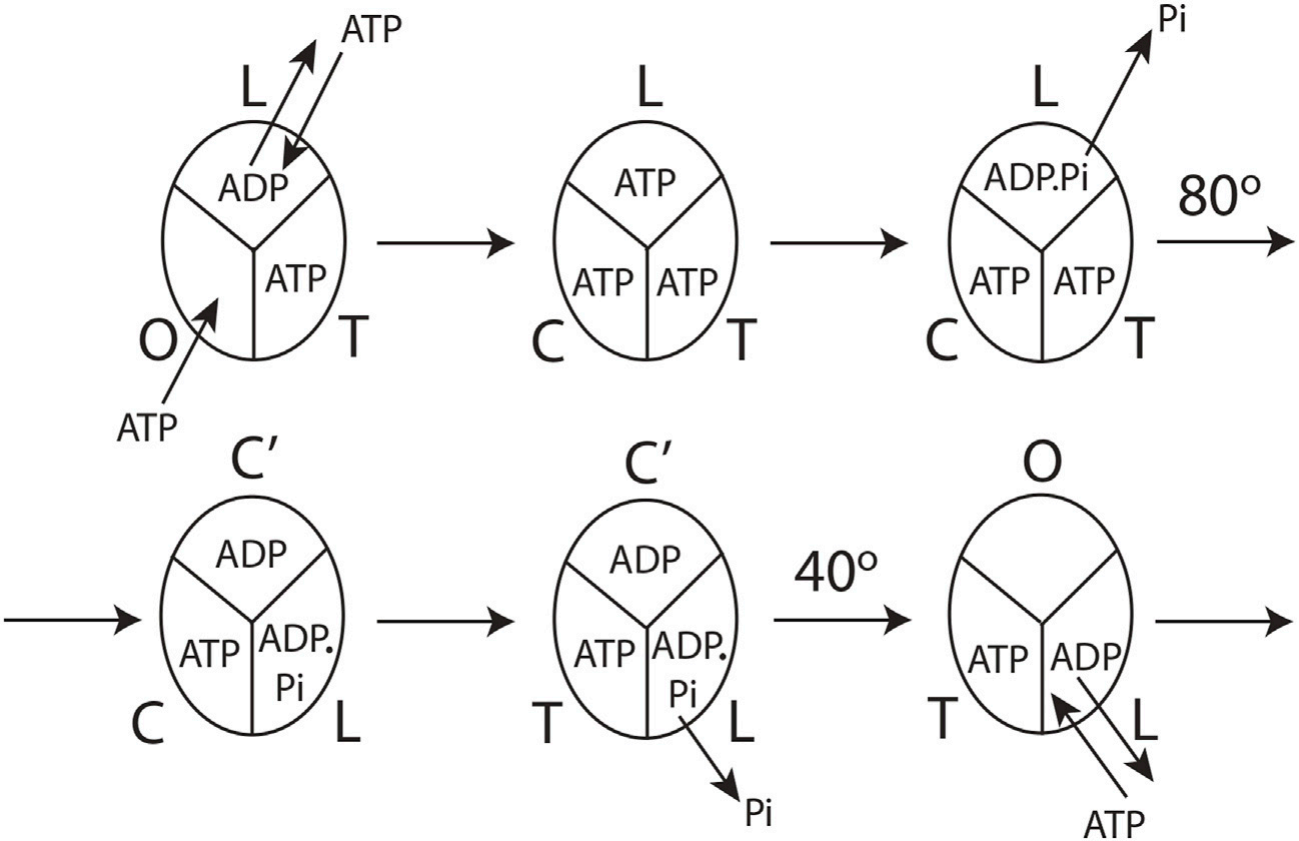
Model for steady-state multisite hydrolysis of ATP by F_1_-ATPase based on experimental data and Nath's torsional mechanism of ATP synthesis/hydrolysis and the unified theory (Nath, 2002; Nath, 2008; Nath and Nath, 2009; Wray, 2015; Mehta et al., 2020). The three β-catalytic sites of the *Escherichia coli* enzyme or the enzyme from thermophilic bacterium are depicted. The system is viewed from the F_1_ side. T represents the catalytic site of highest affinity for MgATP (site 1); L represents the catalytic site of intermediate affinity (site 2); O represents the site of lowest affinity (site 3); Cʹ stands for the conformation adopted by a closed catalytic site (which could even be half-closed, i.e., C) relative to the open (O) site. The diagram is drawn to represent steady-state V_max_ hydrolysis at high (∼mM) concentrations of ATP, i.e., when there is sufficient ATP to fill all three catalytic sites before the rotation of the top of the γ-subunit. The diagram can be easily adapted to a possible scenario at intermediate (micromolar) concentrations of ATP when site 3 is filled after the 80^o^ rotation of γ_top_, already described cogently (Nath, 2008), or, for that matter, at any instant of time during or after activation of the 80^o^ rotary step of γ_top_ by elementary chemical events occurring in site 2. However, site 3 can only adopt a completely closed conformation or tight (T) conformation after the occurrence of the ATP cleavage reaction step in (the new) site 2 upon undergoing a site 1 to site 2 (T → L) transition due to 80^o^ rotation of γ_top_, and after the ε-subunit has rotated clockwise and its interactions with site 3 (O) have been broken, as explained by Nath (2008). The model is described in detail in Section 3.4.

The detailed molecular mechanism of V_max_ ATP hydrolysis by F_1_-ATPase (Nath, 2008) can now be refined to make it consistent with our experimental observations in Section 3.1 and the mechanistic implications and physical principles of biological energy transduction determined in Sections 3.2 and 3.3. Such a mechanism is illustrated in Figure 4.

The molecular mechanism of ATP hydrolysis by F_1_-ATPase (Figure 4) incorporates a key result arising from this experimental study that it is not sufficient to exchange bound ADP in the catalytic site 2 (L) with medium ATP to activate the enzyme and cause an ∼80° primary rotation of the central γ-subunit (in a clockwise sense when viewed from the F_1_ side) in F_1_. The enzyme also needs to hydrolyze the bound ATP in site 2 (L) (exchanged with ADP in the catalytic site) to ADP.Pi. Subsequently, Pi needs to move away and be released from L, as explained in detail in Section 3.3. Hence, a revision of our previous mechanism (Nath, 2008) to include the aforementioned fact is necessary. The exchange of bound MgATP for MgADP in site 2 releases an excess binding energy of 9 kJ/mol in the *E. coli* F_1_-ATPase, i.e., the difference between the binding energy of MgATP in L (36 kJ/mol) and the binding energy of MgADP in L (27 kJ/mol). This 9 kJ/mol energy released weakens the binding of bound Pi formed upon ATP hydrolysis in site 2 to approximately zero (i.e., cleavage of the terminal bond of ATP originally at a bond distance of 0.3 nm (Nath and Nath, 2009)). The effect of ejecting ADP with a certain velocity helps break the ∼9 kJ/mol γ–β_TP_ interactions between γ and site 2. Now, the γ-subunit is free to rotate, and Pi is free to move away from bound MgADP (Section 3.3). As Pi moves stepwise from 0.3 to 0.4 and then to 0.6 nm (Nath and Nath, 2009), it releases a Coulombic repulsion energy of 9 + 9 = 18 kJ/mol. Another 18 kJ/mol is made available as Pi is fired out from 0.6 nm to ∞ and released into the solution. This 36 kJ/mol electrostatic potential energy rotates the top of the γ-subunit by ∼80^o^ relative to the stationary β-subunits with an average torque measuring ∼40 pN nm generated at the β–γ interface at a radial distance of approximately 1 nm from the central axis of the α_3_β_3_ hexamer (Figure 4).

After initiation of the 80^o^ rotation of the top of γ, site 2 (L) changes to a closed conformation Cʹ. Upon the above 80^o^ rotation, the top of γ interacts with the β-catalytic site 1 [T or β_DP-like_ in Nath (2008)] and alters its conformation to loose [i.e., site 2, L or β_TP_ in Nath (2008)]. In other words, the rotation of γ_top_ causes a T → L transition of the β-catalytic site, due to which a ∼9 kJ/mol destabilization (reduction in the binding energy of intermediate bound in the site) occurs. Concomitantly, ATP hydrolyzes to ADP.Pi upon the T → L transition of the catalytic site. Pi, which is bound to L with ∼9 kJ/mol binding energy, is now bound in L with ∼zero binding energy and is, therefore, free to move away. The 0.3 → 0.4 → 0.6 nm movement of Pi away from bound ADP releases ∼18 kJ/mol, which is transmitted from site 2 to site 3 (O or β_E_) along the ε-helix and helps break the ε–β_E_ interaction (e.g., between ε–Ser-108 and β_E_–Glu-381 in the DELSEED loop) (Nath, 2002) along with the ∼27 kJ/mol binding energy of MgATP in site 3 (O or β_E_), as already described in detail by Nath (2008). The open site O or β_E_ closes due to these interactions and relieves the steric hindrance that the open site offered to further rotation of the γ-subunit (beyond 80^o^). If MgATP does not bind to site 3 or only free ATP is present in the external/crystallization medium, then the ATP only binds weakly to site 3, which, therefore, retains its open conformation. The torsional strain in the γ-subunit helps break the ε–Ser-108–β_E_ and ε–Met-138–β_TP_ interactions and hence the bottom of the γ-subunit and the ε-subunit rotate 80^o^ clockwise about the central axis of α_3_β_3_, as viewed from the F_1_ side (Figure 4). Concomitantly, O → T. The two coiled-coil α-helices of the γ-subunit are unwound, thereby relieving the torsional strain. After the 80^o^ rotation of γ–ε is complete, Pi release from the new L (β_TP_) and its movement from 0.6 nm away from ADP to infinity provides the energy for the remaining 40^o^ rotation of the γ- and ε-subunits. Upon this rotation, the interaction of ε with Cʹ changes its conformation to an O-site (open, site 3, the site with lowest affinity for Mg-nucleotide) from which bound MgADP is released. During steady-state V_max_ hydrolysis, the order of conformations that a single catalytic site of F_1_-ATPase passes through is O → T, T → L, L → Cʹ, and Cʹ → O. Looking at the enzyme as a whole, the order of the conformational changes of the catalytic sites during multi-site hydrolysis by F_1_-ATPase is L → Cʹ, followed by T → L, followed by O → T, and lastly, Cʹ → O, which is in accordance with our previous predictions and shown to be the microscopic reversal of the ATP synthesis cycle (Nath, 2008). The cycle (Figure 4) then repeats; other details are given by Nath (2008).

The catalytic cycle for the steady-state V_max_ hydrolysis depicted in Figure 4 is in accordance with biochemical crosslinking studies. These studies inferred from the data that the rotation of the γ- and ε-subunits in *E. coli* F_1_-ATPase is not linked to unisite hydrolysis of ATP at the highest affinity catalytic site 1 (T) but to ATP binding and/or ATP hydrolysis and product release at the second or third catalytic site on the enzyme (i.e., site 2 or 3) (García and Capaldi, 1998). The studies also showed that the effect of covalently crosslinking β–Cys-381 to γ–Cys-87 (i.e., forming the β–γ crosslink) increased the rate of unisite catalysis to that obtained by the cold chase of ATP of the non-crosslinked enzyme (Section 3.1). As β–γ in the biochemical crosslinking studies corresponds to β_TP_ in the X-ray structure of the enzyme in the Mg-inhibited state (Abrahams et al., 1994), we infer that β_TP_ (site 2 or L) is the catalytic site to which ATP binds (in which it subsequently hydrolyzes; see Section 3.2) in the native non-crosslinked enzyme. These events are responsible for rotating γ by 80^o^, changing the conformation of site 1 to site 2, and causing hydrolysis of the bound ATP in the (new) site 2, as shown in Figure 4.

The molecular mechanism shown in Figure 4 also satisfies the fact that V_max_ ATP hydrolysis follows trisite catalysis (Weber and Senior, 2001; Nath, 2002; Nath, 2003), a fact experimentally proven today. This by itself takes it beyond the binding change mechanism, which was necessarily a bisite model (Boyer, 1993). However, over the past 2 decades (Weber and Senior, 1997; Wang and Oster, 1998) and up to the present day (Nakano et al., 2022), ATP binding to site 3 (O) has been repeatedly postulated to cause rotation in F_1_-ATPase. We have pointed out previously that the O-site (site 3) is open and distorted, and the binding energy of MgATP is only 27 kJ/mol (Nath, 2008), which is grossly insufficient energetically to change the conformation of the catalytic site from O to closed (C) and also cause a primary rotation of the γ- and ε-subunits by 80^o^ (Nath, 2008). Above all, as proved in Section 3.3, the ATP binding step is not fully competent to perform the useful work of rotation in the enzyme/molecular machine (Figure 3). It should also be stressed that the detailed mechanism of steady-state multisite ATP hydrolysis by F_1_-ATPase presented here (Section 3.4, Figure 4) is the microscopic reverse of the molecular mechanism of steady-state ATP synthesis by F_O_F_1_-ATP synthase formulated by us in previous publications (Nath, 2002; Nath, 2008; Mehta et al., 2020). The difficult constraint of microscopic reversibility has not been shown to be satisfied by other mechanisms. For these compelling reasons, we consider the mechanism shown in Figure 4 superior to extant mechanisms in the field.

## 4 Discussion

The detailed molecular mechanism shown in Figure 4 and discussed in Section 3.4 was not the result of the application of standard structural or biochemical techniques. It was realized based on a sound knowledge of molecular mechanics (Nath, 2003), protein science and bioinformatics (Nath, 2008), and a unique molecular systems approach (Nath, 2002; Nath, 2006a) developed by creative integration of concepts from physics (Nath, 2017; Nath, 2018c; Nath, 2019a; Nath, 2019b; Nath, 2019c; Nath, 2021a), chemistry (Nath and Nath, 2009; Nath, 2018a; Nath, 2018b; Mehta et al., 2020), biochemistry (Nath, 2010a; Nath, 2010b; Nath and Elangovan, 2011; Nath and Villadsen, 2015; Nath, 2016; Nath, 2020a), biology (Nath, 2020b; Nath, 2022b), physiology (Nath, 2022a), biophysics (Nath, 2021b; Nath, 2021c), pharmacology (Nath, 2022c), pure mathematics (Nath, 2022d), economics (Nath, 2019d), engineering (Nath et al., 1999; Nath, 2002; Nath, 2003), and medicine (Nath, 2019e) spanning 3 decades of research by the author. For perspectives on this approach by other researchers, see Channakeshava (2011), Villadsen et al. (2011), Wray (2015), and Juretić (2022). For a summary of the author’s innovative approach to discovery, see Nath (2006b). However, it is possible to embellish Figure 4 with molecular snapshots from high-resolution X-ray structures (Cingolani and Duncan, 2011; Shirakihara et al., 2015) that were solved several years after the aforementioned molecular interactions of ε–Ser-108 with β_E_–Glu-381 (Nath, 2002; Nath, 2008) and ε–Met-138 with β_TP_ (Nath, 2008), along with their functional roles during catalysis, were postulated.

### 4.1 Structural interpretations and relationship to the F_1_-ATPase catalytic cycle

**FIGURE 5:**
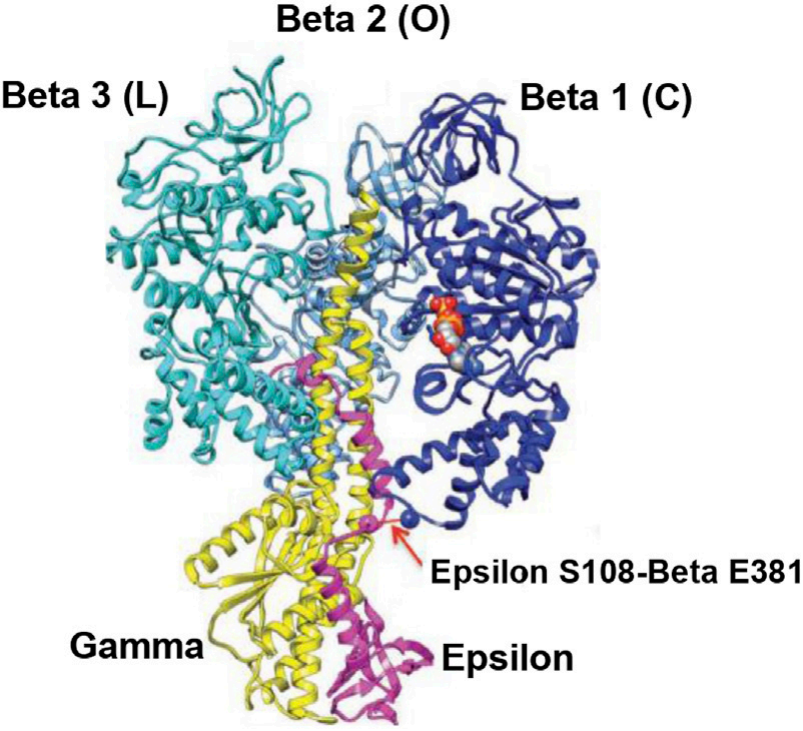
Overall view of the Cingolani–Duncan *Escherichia coli* EF_1_ X-ray structure (Cingolani and Duncan, 2011). A ribbon diagram in the side view with the α-subunits removed for clarity is shown with permission. The extended ε-subunit is shown in pink, the γ-subunit in yellow, and the β-subunits in various shades of blue. The interaction of ε-Ser-108 with Glu-381 of β1 (β_DP-like_) in its half-closed conformation is illustrated by the arrow. The ADP and SO_4_^2–^ bound in β1 are shown as space-fill atoms. However, the X-ray structural snapshot cannot reveal the dynamics of whether the β-catalytic site is moving from an open to a closed conformation, or if the interaction of ε with the catalytic site is helping the site to transition from a closed to an open conformation. Thus, only a detailed molecular mechanism can elucidate the exact role of the ε-subunit and the function of such energy-promoted association of the ε-subunit with the β-catalytic sites and reveal the physiological significance of the movement and dynamics of the small single-copy subunits and their interactions with the β-subunits during catalysis by the F_1_-ATPase/F_O_F_1_-ATP synthase in steady-state ATP hydrolysis/synthesis.

**FIGURE 6:**
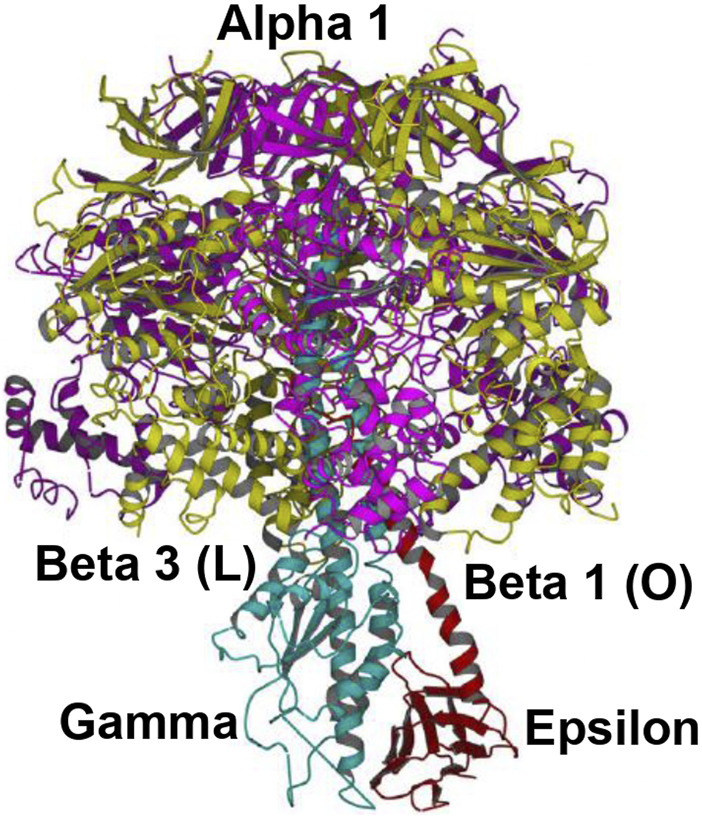
Overall view of the Shirakihara TF_1_ X-ray structure from a thermophilic bacterium (Shirakihara et al., 2015) in side view, drawn with permission. The ε-subunit in its extended conformation is shown in red, the γ-subunit in blue, and the β-subunits β1 and β3 in gold. An intervening α-subunit is shown in magenta. The penetrating C-terminal helix of the ε-subunit into the α_3_β_3_ cavity and its interactions with the β-catalytic sites are shown.

The overall high-resolution X-ray structures of *E. coli* F_1_ (Cingolani–Duncan EF_1_ structure, 3OAA) (Cingolani and Duncan, 2011) and of F_1_ from a thermophilic bacterium (Shirakihara TF_1_ structure, 4XD7) (Shirakihara et al., 2015) are shown in side view as ribbon diagrams in Figure 5 and Figure 6, respectively. The overview of the structures (Cingolani and Duncan, 2011; Shirakihara et al., 2015) in Figures 5 and 6 clearly reveals the interactions of ε–Ser-108 with β_E_–Glu-381 and of the tip of epsilon ε–Met-138 with β_TP_ (Nath, 2008) postulated by Nath's torsional mechanism of ATP synthesis/hydrolysis us previously (Nath, 2002; Nath, 2008), diagrammed in Figure 4, and described in Section 3.4. The following features of the X-ray crystal structures can be related to the detailed mechanism of ATP hydrolysis by F_1_-ATPase.

#### 4.1.1 Cingolani–Duncan EF_1_ structure (Cingolani and Duncan, 2011)

The overall EF_1_ structure shows a highly extended conformation of the ε-subunit (pink) that inserts into the central cavity and interacts with two of the three β-catalytic sites, designated as β1, β2, and β3 (various shades of blue) (Figure 5). The β2 catalytic site is open and contains no bound nucleotide. It does not interact with the C-terminal of the ε-subunit and corresponds to O (β_E_) in panel 1 of Figure 4. The site has not closed or changed its conformation to T (panels 5, 6 in Figure 4), although ε has moved away clockwise (looking from the F_1_-side) because the nucleotide has not bound to the catalytic site.

**FIGURE 7:**
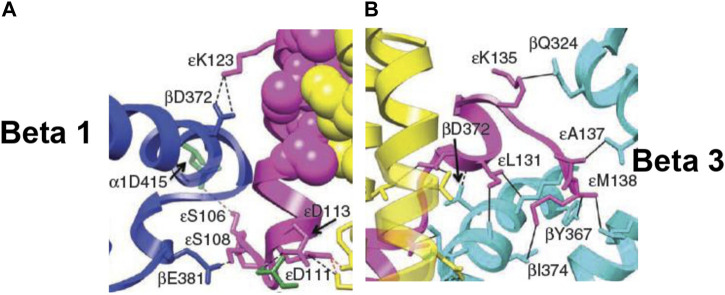
Snapshots from the X-ray structure of EF_1_ (Cingolani and Duncan, 2011), revealing, close-up, the interactions of the ε-subunit with the β-catalytic sites inside the α_3_β_3_ cavity, shown with permission. **(A)** The interactions of the C-terminal domain of the ε-subunit with β1 (β_DP-like_), especially of ε–Ser-108 with β1–Glu-381 [predicted previously by Nath (2002)], are highlighted. **(B)** The interactions of the helix 1-loop-helix 2 motif of the C-terminal domain of the ε-subunit with β3 (β_TP_), especially of the tip of the hook region ending in ε–Met-138 with the nucleotide-binding pocket in β3 [predicted previously by Nath (2008)], are highlighted. Similar interactions of the ε-subunit with β1 (β_DP-like_) and β3 (β_TP_) are seen in the X-ray structure of TF_1_ (Shirakihara et al., 2015).

The catalytic site β1 adopts a half-closed conformation and contains bound ADP (and SO_4_^2–^). ε–Ser-108 interacts with β1–Glu-381, as shown in Figure 5 and visualized by the X-ray structure in the close-up view of Figure 7A. The β1-site is akin to β_DP_ (Nath, 2008) or Cʹ (closed) in Figure 4, also labeled as C in Figure 5 [half-closed with reference to the open site O; note that half-closed, three-fourths closed, etc. mean closed with respect to O in a continuum of conformations (Nath, 2002), of which a frozen snapshot has been captured by the structure]. The N-terminal of the ε-subunit has rotated clockwise from β2, and its C-terminal has started to interact with β1; however, β1 has not yet fully opened due to these interactions. Thus, the site has not yet been converted to O (site 3).

Figure 5 visualizes the interactions of the ε-hook with β3, and Figure 7B depicts these interactions in detail, as seen in the Cingolani–Duncan EF_1_ structure (Cingolani and Duncan, 2011), including the interactions of the helix tip ending in ε–Met-138 with β_TP_ (L) postulated previously (Nath, 2008) and discussed in Section 3.4. β3 adopts a closed conformation but contains no bound nucleotide (ADP). The catalytic site β3 is akin to β_TP_ or L in panel 6 of Figure 4 after hydrolysis and release of Pi. The X-ray structure (Cingolani and Duncan, 2011) is a snapshot before ε-hook has broken its interactions with β3 (L or site 2) and rotated clockwise (looking from F_1_) toward β2 (Section 3.4). In that sense, it has not “rotated farthest in the direction of ATP hydrolysis,” as previously postulated (Cingolani and Duncan, 2011). This conclusion is also validated by the fact that β1 has not yet adopted an open conformation in the structure as noted previously, and as expected in our interpretation, if the structure had rotated farthest in the ATP hydrolysis direction and had therefore corresponded exactly to the 0^o^/120^o^ ATP-waiting state.

Finally, single-molecule rotational studies on various F_1_ species (Kato et al., 1997; Konno et al., 2006; Watanabe et al., 2015) have revealed that the presence of the ε-subunit arrests the ATPase cycle at the catalytic dwell angle of ∼80^o^, which contradicts the assignment of the structure (Cingolani and Duncan, 2011) as depicting an ATP-waiting dwell state of 0^o^/120^o^. A possible reason for this discrepancy could be that the assignment was primarily based on structural alignment by a γ-core method that employed 99 Cα atoms (Shah et al., 2013). However, such assignment is dependent on the choice of the number and distribution of amino acid residues in the γ-subunit. In particular, residues at the bottom of the γ-subunit in structures of F_1_ display considerable angular play. One must be careful before making a unique superposition, as also pointed out by the group that solved the X-ray structures (Rees et al., 2012).

Thus, the crystal structure captures a state of the F_1_-ATPase closer to (but not exactly at) the 0^o^ (or rather 120^o^) ATP-binding dwell than the three-nucleotide filled Leslie–Walker structure, 1H8E (Menz et al., 2001) that traps a post-hydrolysis, pre-product release conformation at θ∼80^o^ (Nath, 2008).

#### 4.1.2 Shirakihara TF_1_ *Bacillus* PS3 structure (Shirakihara et al., 2015)

This high-resolution thermophilic TF_1_ *Bacillus* PS3 X-ray crystal structure (Shirakihara et al., 2015) (Figure 6) is even closer to the 0^o^ (or 120^o^) ATP-binding dwell state compared to the Cingolani–Duncan structure. The site β2 is as described in Section 4.1.1. As to β1, unlike in the Cingolani–Duncan EF_1_ structure (Cingolani and Duncan, 2011), β1 adopts an open conformation (O) with no bound nucleotide. The N-terminal of the ε-subunit has rotated clockwise from β2 (viewed from F_1_) and lies close to the β1-catalytic site. The ε–Ser-108 interacts strongly with DELSEED of β1. Thus, the closed site here has been converted to O by the interaction of the C-terminal of ε with β1 (Nath, 2002; Nath, 2008). The remaining aspects are described in Section 4.1.1. The TF_1_ β3 contains bound ADP, adopts a closed conformation, as after Pi release, and is analogous to L or β_TP_ (Figure 4, Section 3.4). The ε–C-terminal residues 125–130 interact strongly with β3 (Shirakihara et al., 2015).

The overall state of the enzyme is like panel 6 in Figure 4, except that T is open as the nucleotide has not bound to it. Thus, this structure is the closest among the solved high-resolution X-ray structures to the true resting/ground state of the enzyme (Section 3.4).

#### 4.1.3 The Leslie–Walker MF_1_ structure (Menz et al., 2001)

As explained previously (Nath, 2008), the 1H8E Leslie–Walker structure (Menz et al., 2001) with nucleotide bound in all three catalytic sites captures a metastable post-hydrolysis, pre-product release state of the mitochondrial MF_1_, trapped at an angular position of ∼80^o^. It should be noted that the half-closed site (i.e., closed with respect to the open β_E_ (O) site in the 1BMF MF_1_ structure (Abrahams et al., 1994)) had been anticipated several years earlier by Nath et al. (2000) and Nath and Jain (2000) before the Leslie–Walker structure (Menz et al., 2001) revealed the existence of such a closed conformation, reviewed by Nath (2002) and Wray (2015) and communicated personally to this effect by Dr. Andrew Leslie to the author (Leslie, 2006).

#### 4.1.4 The Sobti et al. Cryo-EM structures (Sobti et al., 2016; Sobti et al., 2019)

The aforementioned views are corroborated by the recent moderate-resolution cryo-EM structures of Sobti et al. (2016) and Sobti et al. (2019). Their structure of detergent-solubilized *E. coli* ATP synthase at the 0^o^/120^o^ ATP-waiting dwell in the absence of added nucleotides or Pi (Sobti et al., 2016) revealed a conformation of the β-catalytic sites, very similar to that observed in the Shirakihara *Bacillus* PS3 structure (Shirakihara et al., 2015). In particular, the structure showed a highly extended conformation of ε and an open conformation of β1 without bound MgADP or Pi, as in Shirakihara et al. (2015) and unlike Cingolani and Duncan (2011). The important point is that the mechanism proposed in Figure 4 after a Cʹ-site has converted to an O-site (panel 6) is consistent with the observation of an open β1 site in the cryo-EM structure of Sobti et al. (2016) at the 0^o^/120^o^ ATP-waiting dwell.

The addition of mM MgATP to the *E. coli* F_O_F_1_ ATP synthase enzyme led to major changes in the site conformations at the 0^o^/120^o^ ATP-waiting dwell visualized in a subsequent cryo-EM structure (Sobti et al., 2019). Now, all three β-catalytic sites contained bound nucleotide, the second helix of the ε-subunit at its C-terminal showed an intermediate half-up state, and above all, the site β1 adopted a closed conformation. The mechanism in Figure 4 and Section 3.4 is consistent with these observations; see panels 1–2/3 containing the O to C transition of a catalytic site before the 80^o^ rotational sub-step of γ.

#### 4.1.5 Difficulties with previous mechanistic interpretations and consistency of the interpretations of Nath's torsional mechanism of ATP synthesis/hydrolysis with structural and biochemical observations

A major difficulty with the mechanistic interpretations of Cingolani and Duncan (2011) and Shirakihara et al. (2015) lies in the fact that they consider the ε–β interactions as inhibiting or preventing the conformational changes of the catalytic sites β1 and β3. This view can be justified if rotation is blocked from occurring in F_O_F_1_. However, in our interpretation, for function with its driving force (i.e., in the presence of ion gradients during ATP synthesis or ATP during hydrolysis by F_O_F_1_-ATPase/F_1_-ATPase), the dynamic movement of the ε-subunit and its interactions with β1 and β3 enable the finely tuned β-conformational changes in the process of ATP hydrolysis. The energy transmission required for continued rotation and the progression of the catalytic cycle is funneled to the β-catalytic sites *via* the minor single copy γ- and ε-subunit interactions (Figure 4). Hence, we considered them minor subunits with major roles in catalysis by F_1_-ATPase.

In summary, the large-scale movements of the γ- and ε-subunits described previously are of physiological relevance to ATP hydrolysis/synthesis. These conformational changes of the single copy γ- and ε-subunits and their interactions with the β-catalytic sites reflect functionally distinct intermediate states that are absolutely essential for catalysis by F_1_-ATPase.

Other difficulties exist. The Cingolani–Duncan mechanism requires the dissociation of MgADP and Pi and postulates a rotation of γ by ∼40^o^ before the C-terminal helix 2 of the ε-subunit can switch from its highly extended state and escape from its inserted position in the central cavity (Cingolani and Duncan, 2011). However, an open conformation of β1 without bound nucleotides is observed at this position in the structures of Shirakihara et al. (2015) and Sobti et al. (2016), and the aforementioned interpretation and mechanism are belied by the observation of a closed β1 with bound ADP and sulfate (Cingolani and Duncan, 2011). The molecular mechanism of Figure 4 and the proposals within Nath's torsional mechanism of energy transduction and ATP synthesis/hydrolysis satisfy the requirement of a rotational sub-step—different from the ∼40^o^ sub-step—for the transition of ε from its fully extended to its half-up/down state.

The torsional mechanism is also consistent with the rotational data obtained from single-molecule recordings (Kato et al., 1997; Konno et al., 2006; Watanabe et al., 2015) that the presence of the ε-subunit pauses the F_1_-ATPase at the 80^o^ catalytic dwell angle. This fact is difficult to reconcile with previous mechanistic interpretations (Cingolani and Duncan, 2011).

The known non-competitive behavior of ATP *vis-à-vis* ε in *E. coli* F_1_-ATPase (Sternweis and Smith, 1980; Weber et al., 1999) imposes another difficult constraint, which is not addressed/supported by previous proposals (Cingolani and Duncan, 2011). Since the extended-up state of the ε-subunit is only observed in the absence of ATP, the implication suggests that nucleotide binding to an alternate catalytic site is required to release the extreme C-terminal domain of the ε-subunit. However, MgATP binding to the site/s *per se* cannot relieve the so-called inhibition by the ε-subunit and release the subunit to its half-up/down conformation, in which rotation is permitted, to account for the non-competitive nature of the process. The molecular mechanism shown in Figure 4 and Section 3.4 that incorporates key elements of the proposals of the torsional mechanism (Nath, 2008; Nath and Nath, 2009) is miraculously able to satisfy these very difficult enzymological constraints.

The interpretation of Shirakihara et al.’s (2015) structure, i.e., that functional rotation can be achieved with the C-terminal domain of ε remaining in its extended-up state, is contradicted by the observation of the half-up mobile state of the ε-subunit in the cryo-EM structure of Sobti et al. (2019) in the presence of excess MgATP. The presence of the mobile, almost horizontal helix 2 in an intermediate half-up state of the ε-subunit in which rotation is possible in the enzyme—without the necessity for the ε-subunit to adopt a completely down compact state in which helix 2 is neatly packed between helix 1 and the N-terminal domain of ε—is also consistent with trypsin cleavage studies (Mendel-Hartvig and Capaldi, 1991; Wilkens and Capaldi, 1998), as explained previously (Cingolani and Duncan, 2011). This half-up conformation of ε would allow cleavage by trypsin of the exposed ε-helix 2 while keeping its helix 1 anchored to the γ-subunit and permits ε–Ser-108 in the intervening loop between the two ε-helices to interact with the DELSEED of β1 during functional rotation. The C-terminal helix 2 of ε can re-insert itself into the central cavity and interact with site 1 (T) in a subsequent step before it changes its conformation to site 2 (L), as explained by the torsional mechanism (Section 3.4).

This is not to say that the cryo-EM structures have led to the correct or definitive molecular mechanism of ATP hydrolysis or have always provided meaningful mechanistic insights. In their most recent cryo-EM study, Sobti et al. (2021) performed a snapshot analysis on a slowly hydrolyzing βE190D mutant from the thermophilic bacterium *Bacillus* PS3 ATP synthase under different experimental conditions. However, the authors proposed a considerably different model of rotation in which ATP binding drives the initial 80^o^ rotation and ATP hydrolysis drives the subsequent 40^o^ rotation in F_1_-ATPase. Based on the present biochemical results and our previous work on the torsional mechanism (Nath et al., 1999; Nath et al., 2000; Nath and Jain, 2000; Nath, 2002; Nath, 2003; Nath, 2008; Nath and Nath, 2009; Mehta et al., 2020; Nath, 2021a), we consider the molecular mechanism depicted in Figure 4 and discussed in Section 3.4 to be the right mechanism of steady-state ATP hydrolysis by F_1_-ATPase.

### 4.2 Biochemical consequences

#### 4.2.1 Angular positions of ATP binding, ATP cleavage, Pi release, and ADP release in F_1_-ATPase

The molecular mechanism of ATP hydrolysis by F_1_-ATPase formulated here has several important biochemical consequences. Looking at a single catalytic site, MgATP binds to O (site 3 or β_E_) at 0^o^, which becomes T (site 1 or β_DP-like_) (Nath, 2008) after the ε-subunit moves away during the 0 → 120^o^ rotation of γ–ε. The bound MgATP is hydrolyzed at 200^o^ due to the conformational change from β_DP-like_ to β_TP_ (site 2), owing to a T → L transition of the site. Pi is then released from L at 200^o^, leading to a 40^o^ rotary sub-step (Figure 4, Section 3.4). The ADP unbinds from L at 240^o^ and is fired out because the ADP is *displaced* by medium ATP, which now binds in L (ligand substitution). However, the L-site is meant for ADP.Pi. Therefore, ATP immediately hydrolyzes in site 2 (L), following which Pi is released (“unisite” catalysis in site 2), which gives energy for the 80^o^ rotation sub-step. The L-site now changes to a closed site. The interaction of the C-terminal of the rotated ε-subunit with the closed site induces a conformational change of the catalytic site to its open (O) conformation from which the bound MgADP is unbound and released. A new MgATP binds to O, and the cycle repeats.

In summary, the elementary chemical processes and the angular position at which they occur during ATP hydrolysis by F_1_-ATPase that are consistent with our cold chase experiments with promoter ATP and its analysis are as follows: ATP binding, 0^o^; ATP bond cleavage, 200^o^; Pi release, 200^o^; and ADP release, 240^o^.

The aforementioned correlation of the timing of elementary chemical processes in F_1_-ATPase with rotary angle agrees with the latest biochemical study (2023) in which Nishizaka and coworkers generated a hybrid F_1_ consisting of one mutant β and two wild-type βs in thermophilic *Bacillus* PS3. The enzyme carried a β(E190D/F414E/F420E) mutation, which caused extremely slow rates of both ATP cleavage and ATP binding that enabled unequivocal determination of the angular position of the ATP cleavage reaction (200^o^) after ATP binding at 0^o^ (Hasimoto et al., 2023). However, these authors were not able to decipher the entire mechanism from such experiments, and above all, they could not explain in detail the how and why of the mechanism in F_1_-ATPase.

Our proposed mechanism of ATP hydrolysis is also consistent with our other biochemical findings on mitochondrial F_1_, for instance, those shown in Figure 2, that the ratio of bound ^32^Pi to that of bound ^32^Pi and bound [γ-^32^P]ATP remains constant at approximately 0.333 under various conditions. This result implies that Pi is not bound to more than one of the three catalytic sites on the enzyme at any time. This observed distribution between the bound product and bound substrate is difficult to accommodate using other models.

#### 4.2.2 Mechanism of ATP hydrolysis by the α_3_β_3_γ subcomplex of F_1_

The model of Figure 4 can be readily adapted to explain ATP hydrolysis by the simpler α_3_β_3_γ subcomplex of F_1_ (Adachi et al., 2007). Note that the reverse extrapolation from the mechanism of the α_3_β_3_γ subcomplex to that in the complete F_1_ is non-trivial (i.e., from greater symmetry to greater asymmetry) and cannot be termed scientific as per systems theory (Nath, 2008). In the absence of the ε-subunit, “the identity of the catalytic sites is altered compared to intact F_1_ or F_1_F_O_” and the “O-site exhibits properties, especially of nucleotide binding affinity, akin to that of the C-site,” as explained earlier (Nath, 2008). In terms of the discussion in this article, we can say that the asymmetry conferred by the ε-subunit to site 3 (O) in the intact/normal F_1_ or F_O_F_1_ is lost in the absence of the ε-subunit. Hence, the O-site behaves like a closed site (C), and we can start the catalytic cycle for the α_3_β_3_γ subcomplex from the state sketched in panel 3 of Figure 4.

Starting from panel 3 in Figure 4, we can follow the catalytic cycle of the α_3_β_3_γ subcomplex seen as a whole as it progresses from panel 3 to panel 6 in Figure 4, as L → Cʹ, T → L, C → T, and Cʹ → C (instead of Cʹ → O in the presence of the ε-subunit). The ADP–ATP exchange readily occurs in Cʹ. Hence, the Cʹ-site containing bound ADP (as in panel 5 of Figure 4) is displaced by medium ATP so that the new closed site (C) now contains bound ATP, which cannot hydrolyze in C. In terms of nucleotide exchangeability properties of the catalytic sites in α_3_β_3_γ, T contains tightly-bound non-exchangeable ATP, L can exchange bound ADP for bound ATP whose terminal P_β_–O–P_γ_ phosphoanhydride bond can be cleaved in the L-site by ATP hydrolysis in L, while Cʹ engages in ADP–ATP exchange but the closed site containing bound MgATP (C) cannot be hydrolyzed in C. However, in addition to the L-site, the C-site needs to engage in the ADP–ATP exchange with the medium as otherwise inhibitory MgADP will remain bound in it, and the enzyme shall display its characteristic MgADP inhibition. Therefore, the catalytic cycle of the subcomplex will be arrested at an intermediate angular position of ∼80^o^, as the site does not contain bound MgATP that can hydrolyze, release Pi, and carry out the 40^o^ rotation sub-step to complete the 120^o^ cycle when the site’s conformation changes from Cʹ to C to T to L.

Looking at the subcomplex as a whole, the catalytic cycle of ATP hydrolysis by α_3_β_3_γ can display considerable variability and differences from the coupling scheme of Section 3.4 for ATP hydrolysis by the complete F_O_F_1_ or F_1_-ATPase, for reasons that we shall discuss in the following section. It also explains the difficulty in establishing by single-molecule recordings the relative timing of the various catalytic events, particularly the timing of ADP release. It was impossible to simply add ADP in the medium, as in the experiments on α_3_β_3_γ with phosphate, because of the generic insidious phenomenon of MgADP inhibition (Hirono-Hara et al., 2001). Resolving the angle of ADP release during rapid stepping rotation of α_3_β_3_γ was technically beyond the resolution of even ultra-fast video recording rates. However, various groups in Japan developed innovative imaging approaches to address the aforementioned problem and to resolve the sub-steps during rotation. For instance, Shimabukuro et al. (2003) used the ATP analog ATP-γS whose cleavage on the enzyme was slow, as a result of which the catalytic dwell at 80^o^ was extended to ∼70 ms. The use of fluorescent Cy3-ATP in conjunction with the slowly hydrolyzing ATP-γS at nanomolar concentrations (∼60 nM) allowed the 120^o^ step to be clearly resolved into 80^o^ and 40^o^ sub-steps (Adachi et al., 2007).

Based on the aforementioned single-molecule imaging approaches, Nishizaka et al. (2004) showed that the α_3_β_3_γ subcomplex releases ADP in a 120^o^ step between 240^o^ and 360^o^. Adachi et al. (2007) further narrowed this range and showed that ADP release occurs between 240^o^ and 320^o^. The angle between the binding of Cy3-ATP and its release as Cy3-ADP was 245^o^ ± 57^o^ (mean ± SD), independent of rotary speed, which was interpreted as ∼240^o^ (Adachi et al., 2007). Careful inspection of the raw data reveals a wide angular spread between ADP binding and ADP release spanning a range from ∼120^o^ to ∼360^o^. What were the reasons for such a wide angular spread for what are presumed to be discrete elementary chemical events?

We can glean the following interesting details from a statistical analysis of the 297 recorded pairs of ATP binding–ADP release counts *versus* angle histogram during single-molecule rotation of α_3_β_3_γ (Adachi et al., 2007). (i) In 69% of the pairs, Cy3-ATP binding occurred at the ATP-waiting dwell at 0^o^ immediately before or after, and almost coincident with an 80^o^ rotational sub-step of the γ-subunit (and occasionally with an unresolved 120^o^ step). On the other hand, release as Cy3-ADP occurred within the angular play of an 80^o^ sub-step (or within the time-frame of an unresolved 120^o^ step) starting from 240^o^. (ii) In 9% of the cases (i.e., 26 pairs), ADP release occurred after 360^o^ rotation or more. (iii) In the remaining 22% (i.e., 66 pairs of binding–release events), angles between ATP binding and ADP release were separated by < 240^o^, or ADP release occurred at 240^o^ without rotation. In most cases in (iii), either binding or release was not synchronous with rotation. These apparently “irregular behaviors” (Adachi et al., 2007) posed a difficult conundrum to explain.

Explanations of the irregular statistics summarized previously based on rare blinking events where fluorescence disappears temporarily or resorts to photobleaching and irreversible destruction of Cy3 fluorescence (Adachi et al., 2007) are highly unsatisfactory. The average time for photobleaching in the aforementioned single-molecule experiments was 56 s, an order of magnitude longer than the time for a rotational step of < 1 s. Behavior (ii) cannot be explained away by the successive binding of two (or more) Cy3-ATP molecules. Justifying the irregular behaviors (ii) and (iii) as arising from “non-major reaction pathways” (Adachi et al., 2007) is not acceptable to a theoretician.

Therefore, what is a satisfactory resolution of the aforementioned mechanistic conundrum in terms of the coupling scheme discussed previously and in Section 3.4? The answer depends on how and when one defines the ATP binding step as occurring with respect to the primary 80^o^ rotation of γ after ADP–ATP exchange and Pi release from site 2. Based on this conception, we can take ATP binding as occurring at 0^o^/80^o^ or between these two angles, giving us an 80^o^ angular distribution for the binding step. Furthermore, which ATP molecule is one considering in that definition? These complexities were not considered previously (Nishizaka et al., 2004; Adachi et al., 2007) but can now be analyzed with the help of Figure 4. If the ATP molecule that binds to C is considered a substrate, then as ATP is expected to bind immediately above a critical concentration (i.e., at *high* medium ATP concentrations) into that site, which is the case shown in Figure 4 (i.e., before the 80^o^ primary rotation of *γ* sets the stage for subsequent catalytic events), we can consider ATP binding to C as occurring at 0^o^. However, especially at low ATP concentrations in the medium, the C-site can be filled after the 80^o^ primary rotation of γ, in which case we can say that ATP binding occurred at an angular position of 80^o^. Similarly, ADP release can occur at 240^o^, after the C → T and T → L transitions (and ATP bond cleavage and subsequent Pi release at 200^o^), or since ADP release is recorded to occur between 240^o^ and 320^o^ in the forced rotation experiments and also during stepping motion (Adachi et al., 2007), we can take ADP release as occurring between 240^o^ and 320^o^, consistent with the aforementioned results from single-molecule studies. This gives us an angle between ATP binding and ADP release that varies between 160^o^ and 320^o^ or even more if unresolved steps of 120^o^, seen in a minority of the single-molecule traces, are also considered. At mM ATP concentrations, we shall observe a mean angle between ATP binding to C and release from L of 240^o^, as found in prior histograms (Adachi et al., 2007).

The aforementioned scheme was applicable to the case of ATP binding to C for hydrolysis by the α_3_β_3_γ subcomplex. However, if we consider the ATP that binds to L/Cʹ after ADP–ATP exchange in L-site (Figure 4), then the angle between ATP binding and ADP release events will increase accordingly, as the ATP bound to Cʹ shall only be released after Cʹ → C, C → T, and T → L transitions of the catalytic site. The elementary chemical processes and the angular position at which they occur during ATP hydrolysis by the α_3_β_3_γ subcomplex in that case are as follows: ATP binding, 0^o^/80^o^ (depending on how and when one defines the ATP binding step as occurring with respect to the primary 80^o^ rotation of γ after Pi release from L, and which ATP molecule one is considering in that definition); ATP bond cleavage, 320^o^; Pi release, 320^o^; and ADP release, 360^o^. Given the angular play and uncertainties in the assignment of the ATP binding and ADP release positions discussed previously, both normal and “aberrant” statistics in (i)–(iii) described previously for the α_3_β_3_γ subcomplex are satisfactorily explained by our coupling scheme. Thus, the conundrum posed in this section is resolved without requiring unnecessary, arbitrary assumptions that are difficult to rationalize.

It is of some consequence to reflect on the reasons why 25 years of technologically advanced, superb state-of-the-art single-molecule studies (Kato et al., 1997; Noji et al., 1997; Hirono-Hara et al., 2001; Shimabukuro et al., 2003; Nishizaka et al., 2004; Sakaki et al., 2005; Konno et al., 2006; Shimabukuro et al., 2006; Adachi et al., 2007; Suzuki et al., 2014; Martin et al., 2015; Watanabe et al., 2015; Kobayashi et al., 2020; Zarco-Zavala et al., 2020; Hasimoto et al., 2023) by peerless experimental groups (see Acknowledgments section) are unable to resolve the mechanistic issues in the field of bioenergetics with finality. The first reason concerns the inherent limitation of available experimental techniques in probing complex biological systems. For example, single-molecule imaging can only record the movement of the central γ-subunit on which the fluorescence/optical probe is bound; it cannot observe and dissect the critical accompanying events occurring in (multiple) catalytic sites of the enzyme. The second aspect (no less important) is the lack of attention to theoretical developments in the field. Nath's torsional mechanism of energy transduction and ATP synthesis/hydrolysis has been available for the past 25 years (Rohatgi et al., 1998; Jain and Nath, 2000; Nath, 2002; Nath and Jain, 2002; Nath, 2003; Nath, 2004; Nath, 2008; Nath and Nath, 2009; Nath, 2010a; Nath, 2010b; Nath, 2017; Nath, 2018a; Mehta et al., 2020; Nath, 2021a; Nath, 2022a), i.e., for the same length of time as single-molecule imaging studies on F_1_-ATPase. If we take the help of such a theory for the interpretation of data and use it as a guide for the design of new experiments, which in turn shall provide a fillip to further theoretical refinement, then a productive synergy shall result, which will greatly accelerate progress in these interdisciplinary fields of research.

To sum up, it is clear that the mechanism of ATP hydrolysis by the α_3_β_3_γ subcomplex of F_1_ is not identical to the mechanism of ATP hydrolysis by the complete F_1_-ATPase enzyme. To a large extent, this occurs because of the lack of the ε-subunit in the single-molecule experiments on the subcomplex. Therefore, inordinate care must be taken not to extrapolate results obtained by single-molecule studies on the α_3_β_3_γ subcomplex—or on the enzyme lacking the full ε-subunit, especially its C-terminus (Keis et al., 2006)—to the complete F_1_, as cautioned by us 15 years ago (Nath, 2008).

#### 4.2.3 Relationship of the proposed mechanism to the single-molecule experiments on bovine mitochondrial F_1_-ATPase by Noji and coworkers (Kobayashi et al., 2020)

Our proposed mechanism of ATP hydrolysis, if interpreted correctly, is consistent with a recent single-molecule study on F_1_-ATPase from bovine mitochondria (bMF_1_) that contains the α_3_β_3_ ring and the γδε complex (compare with Section 4.2.2 on the α_3_β_3_γ subcomplex). In our proposed mechanism [Nath (2008), Section 3.4, Section 4.2.1, Figure 4], as in the experiments on bMF_1_ (Kobayashi et al., 2020), the ATP binding dwell is identified as occurring at 0^o^, and the long dwell at ∼80^o^–90^o^. The long dwell is also the angular position of the catalytic pause/dwell and represents the angular position of the ATP cleavage event in both views. These are also broadly consistent with findings on F_1_ molecules from other species (e.g., EF_1_ and TF_1_).

The differences arise from the postulated identity/cause of the short pause/dwell and its chemical state at ∼20° in bMF_1_, the proposed driving force for the 80^o^/90^o^–120^o^ rotational sub-step, and the order of product release steps as per the two views. In fact, in both views, the short pause/dwell is a Pi dwell, except that in Nath's torsional mechanism of ATP hydrolysis and the unified theory, the short pause originates due to the enzyme activation process in the L catalytic site upon ATP hydrolysis (described at great length already) and the primary rotation of γ that arises from the step-wise Pi movement in the catalytic site away from MgADP (Nath and Nath, 2009) before its release into the surrounding medium. Since this movement of Pi through its exit tunnel after unbinding from its binding site is *quantized* in sub-steps, as formulated quantitatively from first principles in Nath and Nath (2009), a slowing down of any one of the sub-steps during the passage of Pi and before its release into the external medium will be reflected as a pause at an intervening angle between the ATP binding dwell and the catalytic dwell in fast recordings at substrate-saturated high ATP (∼mM), as previously observed (Kobayashi et al., 2020). This pause can be relatively short or long (or remain undetected) compared to the catalytic pause/dwell at 80^o^/90^o^, depending on the passage time of Pi, the resolution of the single-molecule recordings, and the intrinsically stochastic nature of the sub-steps, and can occur at a variable angular position depending on the nature and kinetics of F_1_s from different species. Whatever the variable nature of the angle of the intermediate sub-step, the duration of the pause and its kinetics, or the number of sub-steps, the same mechanism operates in Nath, and the process after activation will cause rotation from the angular position of the binding dwell at θ= 0^o^ to the catalytic dwell position at θ= 80^o^–90^o^ as per Nath's theory. Subsequently, Pi release from the (new) site 2 of F_1_ after hydrolysis of substrate ATP during the catalytic dwell causes rotation of γ-ε from θ= 80^o^–90^o^ to θ= 120^o^ (Nath, 2008).

However, in contrast, Noji and coworkers cannot propose the same driving force for the sub-step rotation from θ= 80^o^–90^o^ to θ= 120^o^ as in Nath's model mentioned previously because the authors have already released Pi at 10^o^–20^o^ in bMF_1_ and driven the sub-step from θ= 10^o^/20^o^–80^o^/90^o^ before the catalytic dwell (in which ATP hydrolysis takes place) has occurred. The authors are, therefore, forced to postulate ATP hydrolysis as the driving force for the θ= 80^o^–90^o^ to θ= 120^o^ sub-step in Kobayashi et al. (2020) and Sobti et al. (2021). Furthermore, their postulated order of product release (ADP followed by Pi) is opposite to that in Nath's model, which proposes an ordered and sequential release process with Pi release followed by ADP release. It should be stressed that the order of product release proposed previously (Kobayashi et al., 2020) contradicts the order determined by X-ray crystallographic studies (Rees et al., 2012).

As a result, the driving force for the first (primary/activation) sub-step is also different between the two models. We note at the outset that the proposal of ATP binding alone (or ATP binding plus ADP release from different catalytic sites) as driving the first rotation step of ∼80^o^ is not supported by the results of cold chase experiments reported in this work (Figures 1 and 2). Moreover, a further difficulty posed by the results of Kobayashi et al. (2020) is that the first rotation step is composed of two sub-steps, first from 0^o^ to ∼10^o^–20^o^ and then (after Pi release) from ∼10^o^–20^o^ to ∼80^o^–90^o^. Therefore, it is problematic to assign one or the other sub-step as arising from ATP binding alone (or to be powered by ADP release in addition to ATP binding) and another as being due to Pi release. The angular distance between the Pi dwell (∼10^o^–20^o^) and the catalytic dwell (∼80^o^–90^o^), measuring ∼60^o^–80^o^, is far too large to be powered by Pi release in bMF_1_. If ATP binding and/or ADP release are postulated to cause the entire step of rotation of ∼80^o^–90^o^, then the function of the Pi release step remains unassigned and unknown, and it appears to have no role in driving the rotation. All these acute mechanistic difficulties are overcome by the alternative model.

Models proposed for ATP hydrolysis by F_1_-ATPase based on single-molecule studies contradict experimental data based on direct, real-time monitoring of catalytic site nucleotide occupancies recorded by Senior’s group (Weber and Senior, 2001; Weber et al., 1993; Weber et al., 1996; Löbau et al., 1998). In these models based on single-molecule experiments, rotation occurs with only two sites occupied by Mg-nucleotide. For example, in the models of Yasuda et al. (2001) and Adachi et al. (2007), postulated for the α_3_β_3_γ subcomplex of TF_1_ from thermophilic *Bacillus* PS3, both the 80^o^ and 40^o^ sub-steps of the rotation of the γ-subunit occur in the bisite mode. In the model proposed for human mitochondrial F_1_-ATPase, all three sub-steps for rotation of γ–ε of 0^o^–65^o^, 65^o^–90^o^, and 90^o^–120^o^, postulated to be driven by ATP binding, Pi release, and ATP bond cleavage, respectively (Suzuki et al., 2014), occur with two sites containing bound Mg-nucleotide. For bovine MF_1_, a bisite mode of catalysis is suggested for rotation of γ–ε for the sub-step from 10^o^–20^o^ to 80^o^ and for the 80^o^–120^o^ sub-step (Kobayashi et al., 2020). Models that propose concerted ATP binding to a site and ADP release from a different site (Adachi et al., 2007; Suzuki et al., 2014; Kobayashi et al., 2020) as driving rotation cannot be trisite. These models are incorrect, given that the operative mode of catalysis during steady-state ATP hydrolysis by F_1_-ATPase is trisite. Similarly, physical models (Mukherjee and Warshel, 2011; Nam and Karplus, 2019; Volkán-Kacsó and Marcus, 2022) proposed for the working of the F_1_ motor are not true trisite models and hence are incorrect. Designating an ATPase mechanism as trisite simply because it alternates between having two and three catalytic sites filled with nucleotide at any time is insufficient and constitutes an imperfect criterion. For a mechanism to be truly trisite, catalysis must occur, and rotation must take place during steady-state V_max_ hydrolysis only when all three catalytic sites are occupied by bound Mg-nucleotide. This condition is satisfied by the model proposed within the torsional mechanism (Figure 4).

Studies that directly monitored nucleotide occupancies of β-subunits proposed true trisite models of ATP hydrolysis by F_1_-ATPase (Weber and Senior, 2001; Weber et al., 1993; Weber et al., 1996; Löbau et al., 1998). However, these models contradict longstanding results from single-molecule recordings (Yasuda et al., 2001; Nishizaka et al., 2004; Adachi et al., 2007; Suzuki et al., 2014; Kobayashi et al., 2020; Hasimoto et al., 2023). For instance, the former studies realized that in a trisite mechanism, ATP binding to site 3 with a K_d3_ value of only ∼100 µM is too weak to provide sufficient energy to drive the 80^o^ rotational sub-step. Hence, the autors proposed that ATP binding to site 3, followed by ATP hydrolysis in site 1 acting in sequence, provides energy for the 80^o^ sub-step of the γ rotation (Weber and Senior, 2001; Senior et al., 2002). The problem for these models is that single-molecule experiments on F_1_ have conclusively shown that ATP hydrolysis takes place in site 1 during the catalytic dwell that occurs *after* the 80^o^ sub-step of the γ rotation (Yasuda et al., 2001; Nishizaka et al., 2004; Adachi et al., 2007; Suzuki et al., 2014; Kobayashi et al., 2020; Hasimoto et al., 2023). Hence, the bond cleavage step in site 1 cannot be invoked to drive the 80^o^ sub-step of rotation.

Models that invoke ATP binding to site 3 as solely or primarily responsible for driving rotation (Wang and Oster, 1998; Oster and Wang, 2000; Yasuda et al., 2001; Nishizaka et al., 2004; Mukherjee and Warshel, 2011; Nakano et al., 2022) are also problematic for the reasons spelled out above and in the last paragraph of Section 3.4. Several other difficulties with proposed models of ATP synthesis and hydrolysis have been discussed previously (Weber and Senior, 2001; Nath, 2002; Senior et al., 2002; Nath, 2008). These inconsistencies and mechanistic problems are eliminated by the model shown in Figure 4.

Enzymological studies designed to test the dependence of steady-state rates of ATP hydrolysis on substrate [ATP] concentrations from sub-micromolar to millimolar, along with the simultaneous assessment of nucleotide occupancies in the catalytic sites of F_1_-ATPase in this concentration range, would greatly help provide further mechanistic insights.

#### 4.2.4 Proposed mechanism and single-molecule studies on human mitochondrial F_1_-ATPase by Yoshida and coworkers (Suzuki et al., 2014) and in other mutants and organisms (Shimabukuro et al., 2006; Zarco-Zavala et al., 2020)

Section 4.2.3 shows how the same mechanism is operative irrespective of whether the F_1_ motor is a six- or nine-stepper. Hence, no new assumptions are required to explain the function of F_1_ motors with an intermediate pause before ∼80^o^, such as human mitochondrial F_1_ (hMF_1_), a nine-stepped motor that has been shown to exhibit a Pi dwell/pause at an intermediate angle of ∼65^o^ (Suzuki et al., 2014). Furthermore, the equal distribution of the Gibbs energies among certain sub-steps of Pi movement selected by Nath and Nath (2009) for a general molecular motor documented an ideal case, which anyhow seems to work quite perfectly for the six-stepped TF_1_ and EF_1_ motors, and for nine-stepped motors with a catalytic pause at ∼80^o^, such as bMF_1_ (Section 4.2.3). A slightly uneven distribution between step 3 and steps (1 + 2) in Table 1 of Nath and Nath (2009) can readily replicate larger angular catalytic dwell positions, for example, in hMF_1_, which has been shown to exhibit the catalytic dwell at 90^o^ (Suzuki et al., 2014). This slightly larger angle ( > 80^o^) at which the catalytic dwell occurs in hMF_1_ could have to do with the different binding affinities of the catalytic sites and the values of the interaction energies of the single copy subunits with the β-catalytic sites in hMF_1_.

The unified mechanism also readily explains the working of three-stepped F_1_ motors without making additional assumptions. Yoshida and coworkers previously identified a mutant TF_1_ that rotates without sub-steps at low MgATP concentration when the ATP binding dwell is several seconds long (Shimabukuro et al., 2006). In this mutant, ATP binding, hydrolysis, and product (Pi) release occur within the same 0^o^ dwell. Such a wild-type motor (PdF_1_) has recently been identified in *Paracoccus denitrificans* (Zarco-Zavala et al., 2020). The behavior of such motors is readily explained because the distance between the α_3_β_3_ surface and the surface of γ (i.e., the interface thickness) determines the magnitude of the average torque produced, τ , and energy conservation determines the rotation angle θ as follows:

$${∆G}_{ATP}^{0^{\prime }}=constant=\tau \theta .$$ (3)

If the magnitude of τ generated at the interface lies below or in the vicinity of a threshold value (approximately ∼30 pN nm), then θ can readily reach 120^o^ in a single step, and a three-stepped 3 × 120^o^ rotary motor results. Since nucleotide exchange, ATP binding, followed by ATP bond cleavage and Pi release occur in the ATP-waiting dwell at 0^o^, the primary rotation of γ_top_ occurs as detailed in Section 3.4, except that rotation does not stop at ∼80^o^ but now continues all the way until 120^o^ and generates high torsional strain in γ (Nath et al., 1999; Nath et al., 2000; Nath and Jain, 2000). Relief of this torsional strain in the γ-subunit enables the bottom of the central stalk to rotate in steps in the compete F_O_F_1_ or in a single step in F_1_ to θ= 120^o^. The relay continues the hydrolysis and Pi release in the next catalytic site as described by the torsional mechanism, and the three-stepped cycle continues.

The aforementioned torsional mechanism naturally explains rotation in three-step F_1_ motors because the same process (after ATP binding and hydrolytic cleavage) of Pi unbinding, its movement away from bound MgADP, and Pi release into the medium contributes energy for driving rotation or performing useful work in molecular motors, irrespective of the angular position of the dwell at which this fundamental process occurs (Nath, 2008; Nath and Nath, 2009).

The three-stepped rotation in PdF_1_ (Zarco-Zavala et al., 2020) and in thermophilic *Bacillus* PS3 mutants (Shimabukuro et al., 2006) is very difficult to explain by other models of ATP hydrolysis, at least in their current form.

#### 4.2.5 Novel predictions

Several novel predictions can be made based on the proposed mechanism and the unified theory of ATP synthesis/hydrolysis. They explain the mechanism of action of various inhibitors of hMF_1_ that have major pharmacological applications. For example, Yoshida and coworkers clearly showed that inhibition by sodium azide blocks rotation of hMF_1_ at θ= ∼65^o^ (Suzuki et al., 2014). This corresponds to the arrest of the primary rotation step of the torsional mechanism and unified theory at ∼65^o^, i.e., before the γ-subunit can reach the catalytic dwell state at 90^o^ in which the second hydrolytic cleavage event can occur. Hence, the conformational relay for multisite hydrolysis (signal transmission) will be irreversibly blocked at θ= 65^o^ by azide, and the enzyme shall exhibit a unique state and nucleotide occupancy of the catalytic sites corresponding to panel 2 in Figure 4, according to Nath's torsional mechanism and the unified theory. Thus, azide inhibition is distinct from the MgADP inhibition that takes place at a different angular position ( θ= 90^o^ for hMF_1_) and occurs by a different mechanism.

The pharmaceutically important case of azide inhibition has been discussed previously. More exotic examples include artificial or fused/hybrid F_1_ motors that combine α, β, and γ subunits of F_1_s from various species for which the generated average torque τ lies above a critical value $\tau_{c}$ (Eq. 3). As a rough estimate, $\tau_{c}$ ≅ 50 pN nm. In such constructs, γ shall rotate only to θ< 80^o^; therefore, rotation shall cease because the γ-subunit is unable to reach the ∼80^o^ position of the catalytic dwell in which subsequent elementary chemical events in another β-subunit can continue the rotation. Hence, the prediction can be made that it should be possible to reconstitute/assemble and find at least a few chimera motors for which continuous rotation will not be detected by the single-molecule rotation assay under the experimental conditions usually employed in such studies.

#### 4.2.6 “Unisite” catalysis by F_1_-ATPase

The present work shows that site 1 (T) contains bound ATP that does not hydrolyze by itself in T. In other words, site 1 contains tightly bound, non-exchangeable MgATP. Hence, “unisite” hydrolysis cannot take place in site 1, contrary to the original view of Cross et al. (1982) and Penefsky (1985). However, the second site contains tightly bound (but exchangeable) nucleotide and “unisite” ATP hydrolysis and Pi release readily occurs in site 2 (L). This is the conclusion we have drawn from our radioactive promoter [γ-^32^P]ATP cold chase experiments (Figure 1; Table 1).

Berden et al. had indeed reached the correct conclusion by biochemical site occupancy experiments that site 1 of the normal F_1_ enzyme does not hydrolyze ATP (Hartog and Berden, 1999; Berden and Hartog, 2000). However, the authors overextended their results to conclude that site 1 does not participate in multisite catalysis. Unfortunately, this logic (in the spirit of Nath (2022a) is correct only if site 1 remains unchanged in conformation during multisite catalysis. If site 1 changes to site 2 owing to rotation of the γ-subunit during the catalytic cycle, as we propose (Figure 4, Section 4.2.1), then (the new) site 2 can hydrolyze ATP, release Pi, and participate in multisite catalysis. In any case, the biochemical experiments of Berden showed that site 2 (L) performs “unisite” ATP cleavage and product release (Hartog and Berden, 1999; Berden and Hartog, 2000). The authors also went on the wrong track by focusing on the non-existence of rotation in F_1_-ATPase (Hartog and Berden, 1999; Berden and Hartog, 2000), which is a proven fact (Kato et al., 1997; Noji et al., 1997; Hirono-Hara et al., 2001; Shimabukuro et al., 2003; Nishizaka et al., 2004; Sakaki et al., 2005; Konno et al., 2006; Shimabukuro et al., 2006; Adachi et al., 2007; Suzuki et al., 2014; Watanabe et al., 2015; Kobayashi et al., 2020; Zarco-Zavala et al., 2020; Hasimoto et al., 2023).

Our conclusion that “unisite” hydrolysis occurs in site 2 (β_TP_) is also in full agreement with the finding of an important recent cryo-EM structural study performed under various reaction conditions (Nakano et al., 2022). However, their proposed mechanism for multisite hydrolysis by F_1_-ATPase in which ATP binding to β_E_ causes 120^o^ rotation of γ-subunit is incorrect and suffers from several deficiencies (Nakano et al., 2022), some of which have been outlined in the last paragraph of Section 3.4.

#### 4.2.7 Cold chase and multisite catalysis by F_1_-ATPase

Section 4.2.6 summarizes the important fact that, contrary to the current dogma, bound ATP does not hydrolyze in site 1. Thus, one has to change the conformation of site 1 to cause hydrolysis of the ATP bound in site 1 (T) (Nath, 2003). However, this change cannot occur without rotation of the γ-subunit, and rotation cannot occur unless site 2 (L) binds and hydrolyzes promoter ATP and releases Pi (cold chase). Moreover, since site 2 contains bound ADP, medium ATP needs to kick ADP off in the catalytic site and bind instead [ADP–ATP exchange (Nath, 2008), i.e., *ligand displacement/substitution*] for activation of the system and initiation of rotation. Hence, one is compelled to postulate the coupling scheme shown in Figure 4 for multisite catalysis in F_1_-ATPase, where sequential participation of catalytic sites in a trisite mode leads to steady-state V_max_ activity.

The filling of multiple F_1_ sites by ligand (MgATP) can be modeled by a simple probabilistic approach. If c is the ligand concentration and $K_{d}$ the dissociation constant of the site for the ligand, then the probability p that the site is occupied by the ligand is given as follows:

$$p=\frac{c}{c+K_{d}}.$$ (4)

The probability that the site is not occupied by bound ligand is given by

$$p=1-\frac{c}{c+K_{d}}.$$ (5)

In principle, the aforementioned equations apply to the multisite catalysis case where any of the three catalytic sites are occupied by bound ligands (represented by 1) or remain empty (represented by 0). The fractions of the enzyme species f that contain or do not contain bound ligands are then determined by the product of the relevant expressions written for each site with its characteristic thermodynamic dissociation constant. The fraction of each species then represents the fractional specific activity of the enzyme $\frac{v}{V_{\max}}$ . Hence, the contribution of each enzyme species to $V_{\max}$ can be quantified in terms of percentages.

If $K_{d1}$ , $K_{d2}$ , and $K_{d3}$ represent the dissociation constants for sites 1, 2, and 3 of F_1_-ATPase, respectively, then the fraction of the enzyme species with all three sites occupied (i.e., in state [111]) is given as follows:

$$f_{111}=\left(\frac{c}{c+K_{d1}}\right)\left(\frac{c}{c+K_{d2}}\right)\left(\frac{c}{c+K_{d3}}\right).$$ (6)

The fraction of species with sites 1 and 2 occupied by MgATP (but with site 3 unoccupied) is given by

$$f_{110}=\left(\frac{c}{c+K_{d1}}\right)\left(\frac{c}{c+K_{d2}}\right)\left(1-\frac{c}{c+K_{d3}}\right).$$ (7)

Dissociation constant values for MF_1_ for the three sites have not been reliably measured. However, we can simulate the system with the $K_{d}$ values for EF_1_. It is known from previous studies that EF_1_ is very similar to MF_1_ in terms of nucleotide exchangeability and other biochemical properties, except that the binding at the lowest-affinity catalytic sites is less tight (Cross et al., 1982; Berden and Hartog, 2000; Hartog and Berden, 1999; Cross and Nalin, 1982). Using conditions of Mg^2+^ in excess of ATP, $K_{d1}$ , $K_{d2}$ , and $K_{d3}$ for sites 1, 2, and 3 measure 0.02, 1.4, and 23 μM, respectively (Weber and Senior, 2001). Use of a lower value of the binding affinity of site 3 for MF1 (K_d3_ ∼100–150 mM) does not alter the results presented in the next paragraph to any appreciable extent.

Using the above $K_{d}$ values at 0.3 μM ATP, we find from Eq. 6 that $f_{111}\approx 2\times 10^{-3}$ , while at 10 μM ATP, $f_{111}\approx 0.25$ . If trisite species are responsible for multisite hydrolysis, this corresponds to a rate enhancement by a factor of ∼125. At 1 mM ATP, $f_{111}\to 1.0$ , implying that the rate enhancement from unisite to multisite conditions is about $1/\left(2\times 10^{-3}\right)$ or 500-fold. A calculation that is more accurate and considers [110] and [100] enzyme species in addition to [111] species is given in Supplementary Section S1. These calculations based on a simple stochastic population model explain all our experimental observations in this context in outline and remarkably model several details of the hydrolysis process (Figure 1; Table 1). We have previously shown that a stochastic kinetic theory based on a master differential equation approach successfully captures the details of oxygen exchange processes occurring at a single catalytic site in the ATP synthesis mode during catalysis by the F_O_F_1_-ATP synthase (Mehta et al., 2020). Hence, our approach is remarkably robust and useful in modeling various related biological energy transduction and catalysis processes.

The aforementioned calculations show that, based on the aforementioned K_d_ values of the three β-catalytic sites, there exists a small fraction of enzyme population with [110] and [111] nucleotide occupancies ([TLO] designates the nucleotide occupancies of tight, loose, and open sites respectively, with 1 standing for a filled site and 0 for an unoccupied site), even at sub-stoichiometric conditions of 0.3 μM ATP and 1 μM F_1_ (Cross and Nalin, 1982). Enzyme molecules in state [110] lead to cold chase and radioactive Pi release from site 1, a single turnover event, recorded by the isotope trap. The small fraction of enzyme molecules in state [110] and [111] yields a slow (∼0.1 s^–1^) rate of ATP hydrolysis. At higher ATP concentrations (super-stoichiometric conditions), events due to cold chase by [110] species are integrated into multisite catalysis (Figure 4). Several turnovers due to [111] populated enzyme species (multisite hydrolysis) by the cycle of Figure 4 provide the ∼10^3^ -fold rate enhancement to ∼100 s^–1^ over “unisite” rates—the so-called positive catalytic cooperativity. In Supplementary Section S1, we start from the 1:3 sub-stoichiometric ATP:F_1_ loading initial condition and simulate F_1_-ATPase enzyme activity for the experimental conditions of Figure 1 and Table 1.

Previously, we explained that the differential affinity of nucleotide binding to the three catalytic sites in ATP synthase does not fundamentally arise from “negative cooperativity of binding,” as proposed by the binding change mechanism (Boyer, 1993), but as per the torsional mechanism is “due to asymmetric interactions of the catalytic sites with the γ and ε subunits” (Nath and Jain, 2000). We explained the above so-called positive catalytic cooperativity to be due to the “mode of functioning of the enzyme itself” (Nath and Jain, 2000) and further owing to “an increase in the fraction of the F_1_ enzyme population containing bound nucleotide in all three catalytic sites with increase in substrate concentration” (Nath, 2003). A 2022 structural work stated that “Assuming that F_O_F_1_ adopts an asymmetric architecture during ATP hydrolysis, the alternating participation of β subunits does not require positive catalytic cooperativity,” (Nakano et al., 2022) which is in line with our previous proposals (Nath et al., 2000; Nath and Jain, 2000; Nath, 2002; Nath, 2003) made more than 20 years ago. Here, we calculated enzyme activity based on a probability-based occupancy model of enzyme catalytic sites, which offers further strong support to the aforementioned conclusion (Supplementary Section S1 of Supporting Material).

The aforementioned results also agree with single-molecule recordings that showed that a single (trisite) rotary mechanism operates for F_1_-ATPase over the entire range of ATP concentration from nanomolar to millimolar (Sakaki et al., 2005).

We have thus shown in Sections 4.1.1–4.1.5 and Sections 4.2.1–4.2.7 that various structural and biochemical observations made in the context of ATP hydrolysis by F_1_-ATPase are logically explained.

#### 4.2.8 Differences between competing mechanisms

We have already discussed the lack of competence of binding energy changes in promoting catalysis by F_1_-ATPase (Section 3.3). Furthermore, Boyer’s binding change mechanism was necessarily bisite (Boyer, 1993). However, excellent data obtained using tryptophan fluorescence quenching by Senior and colleagues (Weber and Senior, 2001; Weber et al., 1993; Weber et al., 1996; Löbau et al., 1998) showed that the ATP hydrolysis activity of *E. coli* F_1_ is fully in accordance with the occupation of all three catalytic sites by the substrate (trisite catalysis) (Nath, 2003), in clear conflict with bisite catalysis proposed by the Boyer model. Another fundamental reason for the inadequacy of the binding change mechanism stemmed from the fact that Boyer mainly considered β–β interactions in developing his model. However, during the formulation of Nath's torsional mechanism of energy transduction and ATP synthesis, which was a trisite mechanism (Nath et al., 1999), we considered the *asymmetric* interactions of the β-catalytic sites with γ and ε of fundamental importance (Table 1 of Nath (2003) (Nath et al., 2000; Nath, 2002), and we developed these aspects further in this work. Finally, the important chemical concept of *ligand displacement* is highlighted by our model. However, this concept was missed or ignored during the formulation of Boyer's binding change mechanism, and a complex double hypothesis of negative binding cooperativity and positive catalytic cooperativity was proposed by it (Boyer, 1993). However, there is no chemical necessity for invoking site–site cooperativity or additional allosteric interactions since ligand substitution (e.g., ADP–ATP exchange at a β-catalytic site (Nath, 2008)) can readily cause activation of the asymmetric enzymatic system. Hence, we do not require these additional assumptions. Therefore, we believe that Nath's torsional mechanism of energy transduction and ATP synthesis/hydrolysis and the unified theory offers a simpler solution to the problem and a more elegant and aesthetically appealing picture of energy coupling, transduction, and catalysis by the F_O_F_1_-ATP synthase (Nath, 2002; Nath, 2008).

## 5 Conclusion

The molecular mechanism of ATP synthesis/hydrolysis has inspired a vast amount of research ever since the discovery of ATP almost a hundred years ago. However, the detailed mechanism of the order of the elementary steps in the multiple catalytic sites of an enzyme and how the chemical and mechanical steps are coupled in biological molecular machines has eluded resolution. The current theory considers the binding energy of ATP as the energy source of mechanical rotation in F_1_-ATPase. This view has been shown to be incomplete and incorrect. Fresh insights into the mechanism are obtained by measuring the maximum extents and kinetic rates of hydrolysis of preloaded bound [γ-^32^P]ATP and promoter [γ-^32^P]ATP in cold chase experiments. Mechanistic implications arising from the experiments and the first law of thermodynamics have been spelled out, and general physical principles for biological free energy transduction have been formulated. The question of how ATP acts as an energy source and performs useful external work has been asked and answered. A molecular mechanism of steady-state trisite ATP hydrolysis by F_1_-ATPase, which is consistent with the formulated physical principles and the body of available biochemical information, has been constructed. Only such a detailed mechanism can truly explain the function of F_1_-ATPase as a molecular energy machine. The detailed mechanism of the 120^o^ catalytic cycle (Figure 4) helps us understand the state of the enzyme captured by the unique X-ray structures of *E. coli* EF_1_ (Cingolani and Duncan, 2011), thermophilic TF_1_ (Shirakihara et al., 2015), and the cryo-EM structures of Sobti et al. (2016) and Sobti et al. (2019) that are close to the 0^o^ (or 120^o^) ATP-binding dwell compared to the Leslie–Walker structures (Abrahams et al., 1994; Menz et al., 2001) that trap a metastable post-hydrolysis pre-product release conformation of the mitochondrial MF_1_ at an intermediate angle of ∼80^o^–90^o^ (Nath, 2008). The key and essential involvement of the ε-subunit in sequential energy transfer to/from the β-catalytic sites in F_1_ and the energy-promoted association and asymmetry-causing interactions of the single copy γ- and ε-subunits with the β-catalytic sites (Nath et al., 1999; Nath et al., 2000; Nath and Jain, 2000; Nath, 2002; Nath, 2003; Nath, 2008), leading to critical functionally important changes in the conformation of the catalytic sites, were highlighted in our detailed mechanism. A mathematical model for the estimation of economics and opportunity cost in choosing between competing theories has also been developed (Supplementary Section S2).

A major achievement of the work has been to provide a biochemical basis for interpreting “unisite” catalysis, cold chase, and their relationship to steady-state multisite V_max_ hydrolysis by F_1_-ATPase. These problems have proved frustratingly difficult to solve since the first discovery of “unisite” catalysis more than 40 years ago (Section 4.2). Probability-based calculations of enzyme species distributions and activity have been performed (Supplementary Section S1) to verify the biochemical theory of “unisite” catalysis, cold chase, and multisite catalysis by F_1_-ATPase that offer further support to the results in Table 1 and Figures 1 and 2.

The formulated general physical principles of free energy transduction [(Nath and Nath, 2009) and Section 3.3] and the proposed detailed molecular mechanism of ATP hydrolysis by F_1_-ATPase (Figure 4 and Section 3.4) have important biochemical consequences (Sections 4.1, 4.2). Several novel predictions of pharmacological importance on the mechanism of action of F_1_ inhibitors have been made. The proposed new concept of energy coupling in ATP synthesis/hydrolysis based on ligand substitution chemistry takes us beyond the binding change mechanism of ATP synthesis/hydrolysis. The working mechanism of nine-stepped (bMF_1_, hMF_1_), six-stepped (TF_1_, EF_1_), and three-stepped (PdF_1_) F_1_ motors and of the α_3_β_3_γ subcomplex of F_1_ is explained by the unified theory without invoking additional assumptions or postulating different coupling schemes. The aforementioned developments have wide ramifications (Sections 4.1, 4.2) for a whole gamut of ATP-hydrolyzing molecular motors in biology.

## Data Availability

The raw data supporting the conclusions of this article will be made available by the authors, without undue reservation.
