# Supplementary material for: Beyond binding change: the molecular mechanism of ATP hydrolysis by F1-ATPase and its biochemical consequences
Source: Front Chem. 2023 May 30;11:1058500. doi: 10.3389/fchem.2023.1058500 (PMC10266426; doi:10.3389/fchem.2023.1058500)
Supplement: Supplementary file 1 [file DataSheet1.PDF]

SUPPLEMENTARY INFORMATION 1 (SI 1)

Beyond Binding Change: The Molecular Mechanism of ATP Hydrolysis by F_1-ATPase and its Biochemical Consequences

Sunil Nath*

Department of Biochemical Engineering and Biotechnology, Indian Institute of Technology Delhi, Hauz Khas, New Delhi 110016, India

SI 1 Enzyme Species Distribution and Activity of F_1-ATPase

Dissociation constant values for MF_1 for the three sites have not been reliably measured. However, we can simulate the system with the K_d values for EF_1. It is known from previous studies that EF_1 is very similar to MF_1 in terms of nucleotide exchangeability and other biochemical properties, except that the binding at the lowest-affinity catalytic sites is less tight [107–110]. Using conditions of Mg^{2+} in excess of ATP, K_{d1}, K_{d2}, and K_{d3} for sites 1, 2, 3 measure0.02, 1.4, and 23 μM respectively [32].

Experiment 1: Hydrolysis of pre-loaded sub-stoichiometric [γ-^{32}P]ATP

[γ-^{32}P]ATP = 0.3 μM, F_1 = 1 μM

BASIS: 100 molecules [γ-^{32}P]ATP, 300 molecules F_1-ATPase

At 0.3 μM [γ-^{32}P]ATP, using **Eqs. (6), (7)** etc. of the main paper, we have

% [100] = 76

% [110] = 16

%[111] = 0.2 (< 1)

Thus 16 moecules of ^{32}Pi are released by hydrolysis.

Promoter ATP = 5 μM. 200 F_1-ATPase molecules are free to be populated by cold chase 5 μM. At 5 μM ATP, we have

% [100] = 18

% [110] = 64

%[111] = 14

Of the 76 F_1 molecules in state [100], 64% of them shall at least be in [11] state, i.e. 48 molecules, and 48 molecules of ^{32}Pi will result. Thus we have a distribution from 16 + 48 = 64 molecules of ^{32}Pi to that when all 76 F_1 are in [110] state eventually at equilibrium, leading to 16 + 76 = 92 ^{32}Pi molecules.

Hence the percent of [γ-^{32}P]ATP hydrolysed will increase from **64% to 92%** (**Figure 1, Table 1**), as found.

Experiment 2: Hydrolysis of cold chase promoter [γ-^{32}P]ATP

BASIS: 100 molecules ATP at 0.3 μM concentration, 300 molecules F_1-ATPase (1 μM), and 1500 molecules of promoter [γ-^{32}P]ATP corresponding to 5 μM concentration

At 0.3 μM ATP, **Eqs. (6), (7)** etc. of the main paper lead to

% [100] = 76

% [110] = 16

%[111] ≈ 0

Cold chase by 5 μM promoter [γ-^{32}P]ATP

% [100] = 18

% [110] = 64

%[111] = 14

In 200 free F_1 molecules, we shall therefore obtain 128 + 28 = 156 ^{32}Pi molecules in one turnover of the enzyme, on the average. Further at least 64% of the 0.3 μM ATP will be

activated, i.e. we obtain an additional 0.64 × 76 = 48 ^{32}Pi molecules, and we shall have 156 + 48 = 204 ^{32}Pi molecules.

Further, we shall have 28 molecules of F_1 (out of a total of 200 free enzyme molecules) that are in the [111] state, on the average. At steady state tri-site conditions, we have approximately 100 s^{-1}/3 × 200 ms/1 s = 6.66 catalytic cycles in 200 ms. Thus we obtain in 200 ms an additional (28 × 6.66) × 6 = 1120 ^{32}Pi molecules, i.e. a total of 1120 + 204 = 1324 ^{32}Pi molecules, of the total 1500 [γ-^{32}P]ATP molecules. This is in agreement with the number of multisite catalytic cycles found in ref. [28]. This corresponds to a % [γ-^{32}P]ATP hydrolyzed of (1324/1500) × 100 = **88%**. This percentage will gradually increase to its equilibrium value of ~95% at longer times, and for higher promoter [γ-^{32}P]ATP concentrations, as found in **Figure 1, Table 1**.

Other values of the dissociation constants alter the kinetics and approach to equilibrium, but do not significantly change the nature or trends of the results obtained above. Hence all the experimental results of our work of F_1-ATPase are explained, in the main, by stochastic-based modeling of enzyme action.

SUPPLEMENTARY INFORMATION 2 (SI 2)

Beyond Binding Change: The Molecular Mechanism of ATP Hydrolysis by F_1-ATPase and its Biochemical Consequences

Sunil Nath*

Department of Biochemical Engineering and Biotechnology, Indian Institute of Technology Delhi, Hauz Khas, New Delhi 110016, India

SI 2 Mathematical Model for an Economics and Opportunity Cost Analysis

Given the imperative need for proposed biological mechanisms to obey physical laws and principles [40, 51] and be consistent with experimental data ([32, 101−103] and **Sections 3.3, 3.4, 4, Figures 1–3**), can we estimate the economic and financial loss incurred due to their contravention by postulated theories for the functioning of the F_O [33–35] and F_1 [7, 15] portions of the ATP synthase? An exhaustive literature search revealed the lack of an economics and opportunity cost analysis for choice of a false scientific theory. A Gaussian distribution for loss may yield a rough estimate; however we expect a log-normal probability distribution function based on analogy with Becker's fundamental analysis of the cost of and investment in human capital [111, 112] as applied to risky choice and investment in a new scientific theory. A mathematical model for quantifying the economics and opportunity cost is developed in this section.

Following the seminal works of Becker [111, 112] and Ben-Porath [113], the life of an individual scientist or worker, i can be divided into two or more periods. In the first period heor she may or may not invest in a risky scientific theory that yields an earning at a later time orphase $Y_{i,}$. Investment $S_{i,}$ in a risky return with a discount r is given by the effective return modeled as $R_t e^{-c}$. Investment decisions in learning more about a new theory are based on the expected effective returns in a future phase. In period 1, risky returns are believed to follow a log-normal distribution with parameters (μ_1, σ_1^2). If individual scientists do not learn about the new theory they enter period 2 with the same set of parameters as in period 1.

However, learning can also be viewed as an investment for an individual that involves incurring a cost D_i in the first period. It is assumed that the gains in learning that accrue in period 2 and persist for a later period 3 even when learning has ceased are related to the amount invested in the risky investment, but learning costs are not. Learning leads to the standard Bayesian alteration of the expected μ and σ for a length of time (history), h [111]. Moreover, learning can ideally reduce the transaction cost parameter, r to zero so that a learned scientist or individual realizes a return of R_t on investment $S_{i,}$.

The utility function for the individual can be written as

$$U_i = \sum_t \beta^{t-1} \frac{C_{i,t}^{1-\gamma}}{1-\gamma} \tag{1}$$

where $C_{i,t}$ is the consumption or cost, β a discount parameter, and γ is a risk aversion parameter. The total earnings $E_{i,}$ in period t is given by the equation

$$E_{i,} = S_{i,t-1} R_t e^{-c_{i,t}} + Y_{i,t} \tag{2}$$

Eq. (2) shows the addition of the earnings $Y_{i,}$ in period t obtained and the returns on investment of previous period investments that accrue. Results for the number density of learners as a function of total earnings in period 2 are shown in **SI 2 Figure 1** by backward induction for an interesting base case of the values of the parameters. The results of **SI 2 Figure 1** show that there is a first minimum value (~1.5) (Region I) in total second period earnings below which there is no learning. Between this value and a second threshold value (~2.0) (Region II), individuals participate in the process, but without learning. Above this second threshold value (Region III) scientists invest in and learn the new theory, and obtain also the greatest total earnings (**SI 2 Figure 1**). The area under Region III, after suitable scaling, may therefore be interpreted as the opportunity cost of not learning and therefore not following the new theory or new developments in a field of research.

The y-axis in **SI 2 Figure 1** can be considered as the number of papers in the field over a long time period. This can be conservatively estimated for the ATP field as an average of ~400 per year, for a time period following the proposal of the old theories (~50 years). The number of papers would be at least a factor of five higher, if related medical fields of ATP such as mitochondrial apoptosis, necrosis etc. are included. The x-axis can then be scaled as the cost incurred per publication, which would vary in the range from US $ 20,000 – US $ 100,000 in

Region III. Thus the economic loss or opportunity cost can be calculated as the area under the curve in Region III of **SI 2 Figure 1**. **This loss works out to an estimated US $ 0.25 billion.**

The model for opportunity costs worked out in this section can be further refined by considering heterogeneities in learning costs, information choice, and in general in human capital, along the lines shown by the economics and financial analyses of Becker [111, 112], Willis [114], Card [115], and Veldkamp [116].

SUPPLEMENTARY REFERENCES

111. Becker GS. Human Capital: A Theoretical and Empirical Analysis. University of Chicago Press, Chicago, USA, 1964.

112. Becker GS. Human Capital: A Theoretical and Empirical Analysis, with Special Reference to Education. University of Chicago Press, Chicago, USA, 3rd ed, 1993.

113. Ben-Porath Y. The production of human capital and the life cycle of earnings. *J Pol Econ* (1967) 75:352−365.

114. Willis RJ. Wage determinants: A survey and reinterpretation of human capital earnings functions. In: Handbook of Labor Economics, O Ashenfelter, R Layard, eds, North Holland, Amsterdam, 1987, pp. 525−602.

115. Card D. The causal effect of education on earnings. In: Handbook of Labor Economics, O Ashenfelter, D Card, eds, 1st ed, Volume 3, Chapter 30, Elsevier, Amsterdam, 1999, pp. 1801−1863.

116. Veldkamp L. Information Choice in Macroeconomics and Finance. Princeton University Press, Princeton, NJ, USA, 2011.

Legend to Supplementary Figures

FIGURE 1 (SI 2) Probability distribution function of normalized density/number vs. total gain or earnings in a future period 2 for an active learning, investment and opportunity cost economic model. The values of the parameters used were: $\beta = 0.97$; $\gamma = 3$; $\mu = 0.07$; $\sigma = 0.15$; $D = 0.08$; $h = 3$; $\sigma_1^2 = 0.15$; $r = 0.025$. The mathematical model is described in **Supplementary Section SI 2**. Region I refers to non-learning and non-participating individuals, Region II to participants but non-learners, and Region III shows total second period earnings of individuals who are active learners and have invested in the new developments in a field.

FIGURE 1 (SI 2)

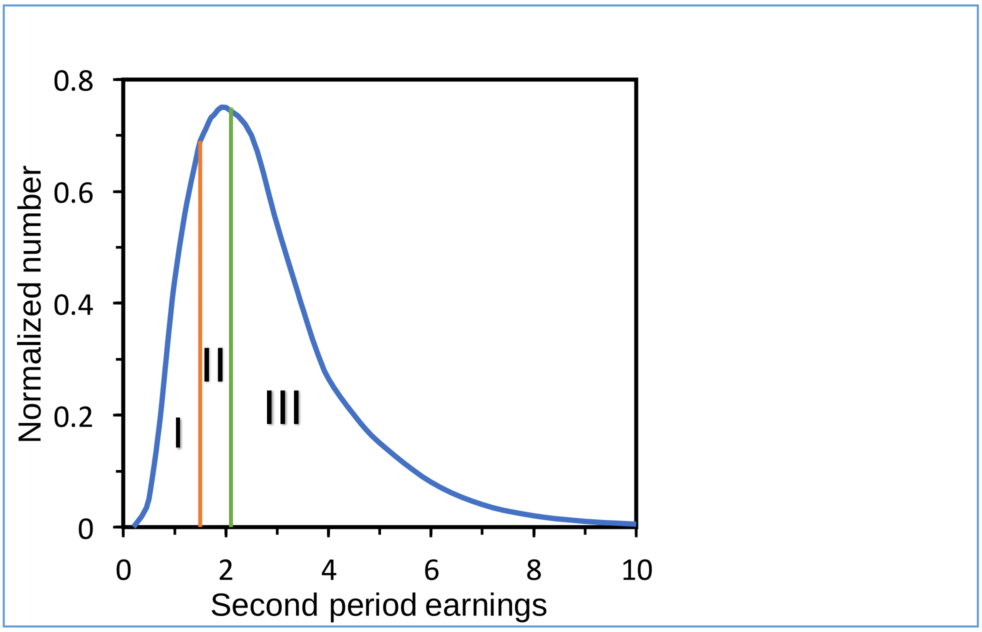

0.8
0.6
0.4
0.2
0
Normalized number
0
2
4
6
8
10
Second period earnings
I
II
III
